# Supplementary material for: Does mandibular bone structure predict subsequent height loss? A longitudinal cohort study of women in Gothenburg, Sweden
Source: BMJ Open. 2023 Jul 4;13(7):e066844. doi: 10.1136/bmjopen-2022-066844 (PMC10391805; doi:10.1136/bmjopen-2022-066844)
Supplement: Author's manuscript [file bmjopen-2022-066844.draft_revisions.pdf]

BMJ Open is committed to open peer review. As part of this commitment we make the peer review history of every article we publish publicly available.

When an article is published we post the peer reviewers' comments and the authors' responses online. We also post the versions of the paper that were used during peer review. These are the versions that the peer review comments apply to.

The versions of the paper that follow are the versions that were submitted during the peer review process. They are not the versions of record or the final published versions. They should not be cited or distributed as the published version of this manuscript.

BMJ Open is an open access journal and the full, final, typeset and author-corrected version of record of the manuscript is available on our site with no access controls, subscription charges or pay-per-view fees (http://bmjopen.bmj.com).

If you have any questions on BMJ Open's open peer review process please email info.bmjopen@bmj.com

BMJ Open

Prospective assessment of mandibular structure as a risk factor for stature shrinkage in women

Journal:	*BMJ Open*
Manuscript ID	bmjopen-2022-066844
Article Type:	Original research
Date Submitted by the Author:	21-Jul-2022
Complete List of Authors:	Gavriilidou, Nivetha N; Karolinska Institute, Department of Dental Medicine<br>Jonasson, Grethe; University of Gothenburg, Department of Behavioral and Community Dentistry<br>Sundh, Valter; University of Gothenburg<br>Rothenberg, Elisabet ; Kristianstad University, Department of Nursing and Integrated Health Sciences<br>Lissner, Lauren; University of Gothenburg
Keywords:	ORAL MEDICINE, Bone diseases < ORTHOPAEDIC & TRAUMA SURGERY, PUBLIC HEALTH

SCHOLARONE™
Manuscripts

I, the Submitting Author has the right to grant and does grant on behalf of all authors of the Work (as defined in the below author licence), an exclusive licence and/or a non-exclusive licence for contributions from authors who are: i) UK Crown employees; ii) where BMJ has agreed a CC-BY licence shall apply, and/or iii) in accordance with the terms applicable for US Federal Government officers or employees acting as part of their official duties; on a worldwide, perpetual, irrevocable, royalty-free basis to BMJ Publishing Group Ltd ("BMJ") its licensees and where the relevant Journal is co-owned by BMJ to the co-owners of the Journal, to publish the Work in this journal and any other BMJ products and to exploit all rights, as set out in our licence.

The Submitting Author accepts and understands that any supply made under these terms is made by BMJ to the Submitting Author unless you are acting as an employee on behalf of your employer or a postgraduate student of an affiliated institution which is paying any applicable article publishing charge ("APC") for Open Access articles. Where the Submitting Author wishes to make the Work available on an Open Access basis (and intends to pay the relevant APC), the terms of reuse of such Open Access shall be governed by a Creative Commons licence – details of these licences and which Creative Commons licence will apply to this Work are set out in our licence referred to above.

Other than as permitted in any relevant BMJ Author's Self Archiving Policies, I confirm this Work has not been accepted for publication elsewhere, is not being considered for publication elsewhere and does not duplicate material already published. I confirm all authors consent to publication of this Work and authorise the granting of this licence.

For peer review only

Prospective assessment of mandibular structure as a risk factor for stature shrinkage in women

Nivetha N Gavriilidou DDS MPH PhD, [a] Grethe Jonasson DDS PhD, [b] Valter Sundh BSc, [c] Elisabet Rothenberg PhD, [d] Lauren Lissner MPH PhD. [e]

[a] Karolinska Institute, Department of Dental Medicine, Institute of Odontology, Karolinska Institute, Sweden. Email: nivetha.gavriilidou@ki.se

[b] Department of Behavioral and Community Dentistry, Institute of Odontology at the Sahlgrenska Academy, University of Gothenburg, Sweden. Email: grethe.jonasson@gu.se

[c.] University of Gothenburg, Dept of Medicine Institute of Medicine, Sahlgrenska Academy, University of Gothenburg, Gothenburg, Sweden. Email: valter.sundh@gu.se

[d] Department of Nursing and Integrated Health Sciences, Faculty of Health Sciences, Kristianstad University, Kristianstad, Sweden. Email: elisabet.rothenberg@hkr.se

[e] School of Public Health and Community Medicine, Institute of Medicine, Sahlgrenska Academy, University of Gothenburg, Sweden. Email: lauren.lissner@gu.se

Corresponding author: Grethe Jonasson, Department of Behavioral and Community Dentistry, Institute of Odontology at the Sahlgrenska Academy, University of Gothenburg, Sweden. Email: grethe.jonasson@gu.se; tel +46 709285671

Short title: Stature shrinkage.

Word count abstract: 258; manuscript: 3035; number of tables: 3; no of figures: 1.

For peer review only - http://bmjopen.bmj.com/site/about/guidelines.xhtml

Abstract

Objective To investigate if the mandibular bone structure could predict future loss of body height in middle-aged and old Swedish women.

Design Prospective cohort study with longitudinally measured heights and radiographical assessments of the cortical bone using Klemetti´s Index (normal, moderately, or severely eroded), and classification of the trabecular bone using an index proposed by Lindh et al. (sparse, mixed, or dense trabeculation).

Setting Gothenburg, Sweden.

Participants A population-based sample of 1003 Swedish women born in 1914, 1922 and 1930, recruited to baseline examination at ages 38, 46, and 54. All underwent a dental examination with panoramic radiographs of the mandible, and a general examination including height measurements on at least 2 occasions.

Main outcome measure Height shrinkage was calculated over three 12-13-year periods (1968-80, 1980-92, 1992- 2005).

Results. Mean annual height shrinkage measures were 0.075cm/year, 0.08 cm/year, and 0.18 cm/year over the 3 observation intervals, corresponding to absolute decreases of 0.9 cm, 1.0 cm, and 2.4 cm. Cortical erosion in 1968 and 1980 significantly predicted stature shrinkage 12 years later. Sparse trabeculation in 1968, 1980 and 1992 also predicted significant shrinkage over 12 or 13 years. Multivariable regression analyses adjusting for baseline covariates height, birth year, physical activity, smoking, BMI, and education yielded consistent findings.

Conclusion. Mandibular bone structure alterations such as severe cortical erosion and sparse trabeculation may serve as proxy indicators of stature shrinkage. Since most individuals visit

For peer review only - http://bmjopen.bmj.com/site/about/guidelines.xhtml

their dentist at least every two years and radiographs are taken, a collaboration between dentists and physicians may open opportunities for risk detection of stature shrinkage.

Keywords. Compact bone; height loss; radiography; trabecular bone.

For peer review only

For peer review only - http://bmjopen.bmj.com/site/about/guidelines.xhtml

Strengths and limitations of this study

- The strengths are the prospective design, the population-based sample, the long follow-up and that our findings can be applicable to many settings since most individuals visit their dentist at least every two years and radiographs are taken.
- The main limitation is that loss of height could reflect different conditions which may make accurate height estimates difficult.
- The participation rates became lower in the later years, and the drop out for different reasons, mortality included, probably differs between age groups, which might impact the mean stature losses.

For peer review only

For peer review only - http://bmjopen.bmj.com/site/about/guidelines.xhtml

INTRODUCTION

Height loss is common in older women. It has been found to be associated with increased morbidity and mortality and it is incrementally more pronounced at ages over 75 years (1-4). Several causes have been identified that lead to loss of height among older adults. Stature shrinkage could indicate age-associated progressive skeletal deformation such as kyphoscoliosis flattening of plantar arch and altered posture (5,6), and/or degenerative processes including osteoporosis, vertebral compression and attrition of intervertebral discs and vertebral fractures (7). These alterations make accurate stature estimation challenging. Comorbidities of major stature shrinkage in older adults include functional impairments such as dependence in daily life activities, cognitive impairment, in comparison with their healthy counterparts, and increased overall mortality risk (1,3). Height loss over 5cm has been shown to predict non-spinal fractures such as hip fractures and mortality independent of vertebral fractures (1-9). In men, health consequences of height loss over 3 cm include increased risk of all-cause mortality and coronary heart disease (8).

Osteoporosis is a widely acknowledged risk factor for future fractures in the elderly. According to the World Health Organization, it is defined as bone density lower than 2.5 standard deviations under that of young women using the bone mineral density of the hip with dual X-ray absorptiometry (DXA) (10). Simple, practical, and relevant techniques are needed for early identification of the onset or extent of skeletal degeneration. Therefore, researchers have used oral variables such as optical density (11), sparse trabeculation (11-13), cortical width (14), and cortical erosion (14-16) for the prediction of osteoporosis and fracture.

Intraoral radiographs are usually an integral part of the routine oral examination. Assessment of mandibular structure, the cortical and trabecular bone pattern may be studied through visual inspection, hence providing a simple screening method for the evaluation of the bone condition. The hypothesis of the present study is that the mandibular bone contains

For peer review only - http://bmjopen.bmj.com/site/about/guidelines.xhtml

sufficient information about the general bone condition to predict future height shrinkage. The objective is to investigate two of the proxies for BMD namely cortical erosion and the coarseness of mandibular trabeculation assessed visually on panoramic radiographs as predictors of stature shrinkage (loss of height) estimated over subsequent periods of 12-13 years.

METHODS

Study population

The Prospective Population Study of Women in Gothenburg, Sweden, is an ongoing longitudinal study initiated in 1968 in a cohort of women aged 38, 46, 50, 54, or 60 years. At that time, a representative sample of women in Gothenburg was identified using the Revenue Office register with respect to the dates of birth. From the full baseline cohort, those born in 1914, 1922, and 1930 were included in the present study sample (flowchart, Figure 1). The original survey included general health and dental examinations, which were repeated over the years. Each survey, started in the fall and completed in the spring of the following year, i.e., 1968-69, 1980-81, 1992-93, and 2004-05, and is hereafter referred to by its starting year. The participation rate in the initial examination was over 90% (17,18). A non-participation analysis of the 24-year follow-up in 1992 showed that the women who declined to participate in the first survey did not differ significantly from participants except for the long-term survival which was lower in the initial refusers. The sample remained representative of the general population in terms of parameters such as blood pressure, weight, height, BMI, waist and hip circumference, serum cholesterol, triglyceride, and urate (18).

Inclusion criteria

In total, 1003 women born in 1914, 1922 or 1930, were included in the present analysis. The inclusion criteria were availability of at least one panoramic radiograph in 1968, 1980, or

For peer review only

For peer review only - http://bmjopen.bmj.com/site/about/guidelines.xhtml

1992 from which it was possible to assess the mandibular cortex erosion and sparsity of trabeculae, and availability of a baseline and at least one subsequent height measure. Results from previous observational studies using the same cohorts have shown that the number of participants should be sufficient for the purpose.

Patient and Public Involvement

No patients involved.

Main outcome

The main outcome variable was stature shrinkage during the 12-13 years following the dental examination. At all examinations, height and weight were assessed by standardized anthropometric measurement techniques. A stadiometer was used to measure the participants' body height (without shoes). Measurement of height was generally performed early in the day. Height loss was calculated as baseline height minus height at follow-up. Thus, a positive value indicated height loss.

Height change was assessed from follow-up examinations 12-13 years later, until year 2005. The exclusion criterion was *increase* in height of at least 2 cm observed during any of these follow-up periods, attributable to probable measurement errors at baseline or follow-up.

There were three 12–13-year follow-up periods namely 1968-1980, 1980-1992, and 1992-2005 where the first period comprised middle-aged women initially aged 38, 46, 54 years, the second period with a subset of these women from ages 62, 70 and 78 years, and the third and the latest period starting at ages 75 and 83 years (no follow-up from 1914 birth cohort) (Figure 1).

Independent and dependent variables

Radiographic assessment of mandibular bone structures was included as part of the dental examination. In the present analysis, the main independent variables were two components of mandibular bone status. On visual inspection of the panoramic radiograph of a mandible, the

For peer review only - http://bmjopen.bmj.com/site/about/guidelines.xhtml

cortical bone was categorized using Klemetti´s Index as normal, moderately, or severely eroded (19). The second component, the trabeculation, was classified using an index proposed by Lindh et al. 1996 as sparse, mixed, or dense (12,13).

Body mass index (BMI) in kg/m^2 was also calculated from these examinations. Baseline variables such as smoking, physical activity, education, and BMI were considered potential covariates that could be associated with mandibular erosion and trabecular pattern, as well as shrinkage (20-23). The survey questionnaire used at the baseline health examination included the following question on smoking habit comprising 6 possible responses: Never smoked (0), Previous smoker but quit in the last 15 years (1), Previous smoker but quit since last year (2), Quit smoking since last year (3), Current smoker without deep inhalation of the smoke (4), and Current smoker with deep inhalation of the smoke (5). This variable was dichotomized to "*No smoking*" (0) including response options 0, 1, 2, and "*Smoking*" (1) including options 3, 4, or 5. The variable physical activity was categorized as (i) almost no physical activity or none (sedentary), (ii) at least 4 hours/ a week of physical activity, (iii) regular physical activity and (iiii) regular intense training and competitions (24). This variable was also dichotomized as: "*physically inactive*" (0) including the first response and "*physically active*" (1) including the latter three choices. Education was considered a proxy for socioeconomic status as proposed by Kaufmann (25) and categorized as pre-high school (up to 9 years); high school with ≤2 years at university; and 3 or more years at university, referred to low, medium, and high level.

Statistical analyses

The Kruskal-Wallis test was used to analyze differences in height loss (stature shrinkage) between the three categories of cortical erosion, whereas Mann-Whitney Two-Sample Test was used for differences between two trabeculation groups. In the analyses, the trabeculation variable was dichotomized into sparse and normal (mixed and dense). For multiple regression

For peer review only - http://bmjopen.bmj.com/site/about/guidelines.xhtml

analysis models, height change was the dependent variable, and the following variables at the start of each period were independent covariates: the bone variables, smoking, physical activity, BMI, education, age (birth year) as well as weight. The significance level was $p<0.05$). Statistical analyses were performed with SAS version 9.2 software (SAS Institute Inc., Cary, NC).

RESULTS

Selected characteristics of the sample are shown in Table 1. Mean age at the four height measurements (year 1968, 1980, 1992, and 2005) were 44.9, 56.9, 68.0, and 78.8 years respectively, corresponding to three periods of height change (1968-80, 1980-92, and 1992-2005).

There was a rising trend in the proportion of women with severe cortical erosion (from 3.2% among the participants in the period 1968-1980, to 11.1% in the period 1980-1992 and up to 49.8% in the period 1992-2005) and sparse trabeculation (from 21.8% among the participants 1968-1980, to 37.2% in 1980-1992 and up to 48.3% in the period 1992-2005). At the same time, the mean height loss accelerated, 0.9 cm, 1.0 cm, and 2.4 cm at the three survey periods, with corresponding annual decreases of 0.075, 0.08, and 0.18 cm/year (Table 1).

Table 1: Descriptive characteristics of the population sub-samples born in 1914, 1922 and 1930 at examination year 1968 followed up across periods 1968-80, 1980-92, 1992-2005.

	**1968**	**1980**	**1992**	**2005**
Age of women born 1930 years (N)	38 (342)	50 (289)	62 (251)	75 (190)
Born 1922	46 (419)	58 (327)	70 (239)	83 (140)
Born 1914	54 (172)	66 (135)	78 (67)	91 (0)[a]
	Total (933)	Total (751)	Total (557)	Total (330)
**Parameters at the end of the period**	**1968**	**1968-80**	**1980-92**	**1992-2005**
Mean age (SD)	44.9 (5.8)	56.9 (5.8)	68.0 (5.4)	78.8 (3.8)
Mean height in cm (SD)	163.9 (6.0)	163.0 (6.1)	162.1 (6.2)	160.4 (6.1)

For peer review only - http://bmjopen.bmj.com/site/about/guidelines.xhtml

Almost no physical activity N (%)	157 (16.8)	221 (29.4)	117 (21.0)	25 (9.3)
Active Smokers N (%)	308 (41.0)	257 (34.3)	115 (20.6)	30 (9.2)
Education level[b]				
Low	630 (67.7)	506 (67.4)	364 (65.4)	209 (63.3)
Medium	251 (27.0)	205 (27.3)	159 (28.5)	93 (28.2)
High	49 (5.3)	39 (5.2)	33 (5.9)	28 (8.5)
Mean body mass index (kg/m^2)	23.7 (3.7)	24.7 (3.9)	26.4 (4.2)	26.2 (4.5)
**Period 1968- 80**				
Mean height Loss (cm (SD))	0.90 (0.89)			
Mean annual decrease in height (cm/year)	0.075			
**Period 1980- 92**				
Mean height Loss (cm (SD))		1.0 (1.21)		
Mean annual decrease in height (cm/year)		0.08		
**Period 1992- 2005**				
Mean height Loss (cm (SD))			2.4 (1.54)	
Mean annual decrease in height (cm/year)			0.18	
**Mandibular parameters at the start of the period**				
Cortical Erosion** *N (%)*				
Mild/None	583 (65.2)	451 (62.9)	175 (33.6)	5 (8.2)
Moderate	283 (31.7)	243 (33.9)	288 (55.3)	128 (42,0)
Severe erosion	28 (3.1)	23 (3.2)	58 (11.1)	152(49.8)
Trabeculation***N (%)*				
Normal	650 (79.4)	516 (78.2)	307 (62.8)	154 (51.7)
Sparse	169 (20.6)	144 (21.8)	182 (37.2)	144 (48.3)

Notes: Cortical bone was categorized using Klemetti´s Index as normal, moderate, or severely eroded and the trabeculation classified into sparse or normal (mixed, or dense). Number excluded based on shrinkage values over period 1968-1980: 4, 1980-1992: 1, 1968-1992: 4, 1992-2005: 5.

[a)]: There were none in this age group in the follow up period 1992-2005.

Not all participants with height measurements had an assessment of cortical erosion and even fewer had trabecular assessment due to edentulism.

[b)]: Education level categories: Low: pre-high school level (up to 9 years), Medium: high school level with ≤2 years at university, High: 3 or more years at university.

Table 2 describes prospective analyses of how dental status at the three age periods predicted stature shrinkage, without covariate adjustment.

Table 2: Stature shrinkage in relation to cortical erosion and trabecular spacing.

**Mandibular bone structure**	**Shrinkage (1968-80)** [a] **N= 751**	**Shrinkage (1980-92)** [a)] **N= 557**	**Shrinkage (1992-2005)** [b)] **N= 330**

For peer review only - http://bmjopen.bmj.com/site/about/guidelines.xhtml

Cortical Erosion	Mean±SD	Mean rate/year	p	Mean±SD	Mean rate/year	p	Mean±SD	Mean rate/year	p
Mild/None	0.84±0.84	0.07	***	0.61±0.928	0.05	***	2.48±1.42	0.19	ns
Moderate	0.99±0.92	0.08		1.04±1.17	0.09		2.21±1.22	0.17	
Severe	1.46±0.75	0.12		1.62±1.15	0.13		2.62±1.86	0.20	
**Mandibular trabeculation**									
Normal	0.88±0.86	0.07	*c)	0.81±1.04	0.07	**d)	2.16±1.26	0.16	**e)
Sparse	1.01±0.99	0.08		1.11±1.15	0.09		2.72±1.82	0.20	

Notes: Assessments at the start year of 12-year periods (1968-1980, 1980-92 and 1992-2005), with statistical significance of the difference between moderate or severe vs no cortical erosion and between normal vs sparse trabeculation. Mandibular cortical bone was categorized using Klemetti´s Index as normal, moderate, or severely eroded and the trabeculation classified into sparse or normal (mixed, or dense).

[a)] Mean shrinkage was estimated from 12- year follow-up period. [b)] Mean shrinkage was estimated from 13-year follow-up period.

***: p<0.001; [c)]: p=0.011; [d)]: p=0.004; [e)]:p=0.002

Loss of height was highest in the groups with severe cortical erosion in the two first periods compared to that of mild erosion, whereas the group with sparse trabeculation had the highest bone loss compared to the normal/mixed group during all three observation periods. On average, the difference in shrinkage between those with mild versus severe erosion was 0.62 cm in the early period, 0.99 cm in the middle period, but only 0.14 cm in the last period (Table 2). In other words, the severe erosion group had greater shrinkage than those with mild erosion of the mandibular cortex.

The difference in shrinking between the group with normal/mixed and that with sparse trabeculation was 0.13 cm in the early period, 0.30 cm in the middle period, and 0.56 cm in the last period. Such values show that, in all the three periods, the sparse trabeculation group presented with more shrinkage than that with normal or mixed trabeculation (Table 2).

Multivariable linear regression analyses in Table 3 confirmed the significant associations between the stature shrinkage and severe cortical erosion during the periods 1968- 80 and 1980-92, but not 1992-2005, after adjusting for covariates as height, age (birth year), physical

For peer review only

For peer review only - http://bmjopen.bmj.com/site/about/guidelines.xhtml

activity, smoking, education, and BMI. Sparse trabeculation showed a significant correlation with shrinkage in all three periods (Table 3).

Table 3: Multivariable regression models predicting stature shrinkage over time intervals 1968-1980, 1980-92 and 1992-2005. Models predicts stature shrinkage among women based on cortical erosion and trabeculation at baseline (1968), after adjusting for covariates height, year of birth, smoking physical activity, education, and body mass index.

	**[a]Shrinkage (1968-80) N= 751**		**[a] Shrinkage (1980-92) N= 557**		**[b] Shrinkage (1992-2005) N=330**	
	Coefficient (Std Error)	P value	Coefficient Std Error	P value	Coefficient Std Error	P value
**Cortical Erosion**	**0.14 (0.06)**	**0.013**	**0.30 (0,09)**	**0.001**	**0.15 (0.15)**	**0.338**
Height at baseline	0.01 (0.01)	0.217	0.02 (0.01)	0.088	0.02 (0.02)	0.156
Birth year	-0.05 (0.01)	0.000	-0.06 (0.01)	0.000	-0.02 (0.02)	0.321
Physical activity	-0.10 (0.07)	0.437	-0.25 (0.12)	0.036	0.10 (0.19)	0.481
Smoking	-0.04 (0.06)	0.517	0.06 (0.11)	0.579	0.34 (0.19)	0.013
Education	-0.01 (0.05)	0.938	0.09 (0.09)	0.299	0.39 (0.15)	0.009
BMI	0.01 (0.01)	0.194	-0.01 (0.02)	0.828	0.012 (0.03)	0.490
Unadjusted	0.21 (0.06)	0.001	0.45 (0.08)	0.000	0.18 (0.14)	0.195
**Sparse trabec.**	**0.17 (0.08)**	**0.030**	**0.24 (0.12)**	**0.021**	0.40 (0.19)	**0.032**
Height at baseline	0.01 (0.01)	0.048	0.02 (0.01)	0.015	0.02 (0.02)	0.359
Birth year	-0.06 (0.01)	0.000	-0.06 (0.01)	0.000	-0.01 (0.02)	0.554
Physical activity	-0.24 (0.07)	0.001	-0.26 (0.12)	0.026	-0.01 (0.26)	0.977
Smoking	-0.04 (0.07)	0.576	-0.07 (0.10)	0.509	0.33 (0.19)	0.076
Education	-0.03 (0.06)	0.553	0.11 (0.08)	0.214	0.36 (0.14)	0.017
BMI	0.01 (0.01)	0.154	0.02 (0.02)	0.248	0.01 (0.02)	0.624
Unadjusted	0.13 (0.08)	0.116	0.30 (0.11)	0.006	0.52 (0.17)	0.003

Notes: Mandibular cortical bone was categorized using Klemetti´s Index as normal, moderate, or severely eroded and the trabeculation classified into sparse or normal (mixed, or dense).
[a)] Mean shrinkage was estimated from 12- year follow-up period, [b)] Mean shrinkage was estimated from 13-year follow-up period.

For peer review only

The significance of the other risk factors for shrinking varied depending on which bone variable was modeled as the main exposure. When adjusted for severely cortical erosion, age was a significant risk factor in the first two periods, physical activity was protective in the middle period, and smoking and high education were both risk factors in the last period, whereas BMI and initial height were not significant. When adjusted for sparse trabeculation,

For peer review only - http://bmjopen.bmj.com/site/about/guidelines.xhtml

age and initial height were risk factors in the first two periods, and physical activity was protective in these two periods. Smoking and BMI were insignificant in all periods and high education was a risk factor in the last period. (Table 3).

DISCUSSION

The present study was conducted among three systematically recruited birth cohorts from the Prospective Population Study of Women in Gothenburg, Sweden. We found that the longitudinal height loss was significantly predicted by mandibular bone parameters such as severe erosion of the cortex and sparse trabeculation. There was a clear increase in shrinkage over the follow-up time, in parallel with an increasing cortical erosion and sparseness of trabeculae. Cortical erosion was the best risk factor for shrinkage in the first two periods when the participants were youngest, and the trabecular sparseness was a better predictor in the last period meaning at the oldest ages.

The strength of this study is that it is based on longitudinally measured heights and assessment of mandibular bone structure across a long follow-up period from a population-based cohort, representing women in Gothenburg, Sweden. Our findings can be applicable to many settings. As mentioned, Swedish and Norwegian women have the highest frequencies of fragility fractures in the world (26); i.e. they probably have sparser trabeculation and more severe cortical erosion than women in other countries, but if for instance Japanese women have severely eroded cortex (15), they may be of risk for height loss as well. Our main finding that cortical erosion and sparse trabeculation predict stature shrinkage builds on previous work in these cohorts showing mandibular bone structure to be a risk factor for future fractures (23,27, 28). Moreover, it was recently demonstrated, using national register data linked to this same cohort, that loss of height over 12 years predicted mortality from all-

For peer review only - http://bmjopen.bmj.com/site/about/guidelines.xhtml

cause and cardiovascular disease (4). Such data is not widely available in prospective epidemiological studies.

However, there are some limitations. Firstly, loss of height could reflect different conditions ranging from postural variations to structural deformations some of which may make accurate height estimates difficult. Secondly, the participation rates became lower in the later years, and the drop out for different reasons, mortality included, probably differs between age groups, which might impact the mean stature losses.

In the first two examination periods, physical activity at the baseline of the study was protective against height loss, which is also in line with other studies (20,29). On the other hand, smoking habits at the baseline had an effect only in the last period where the participants were the oldest, in contrast to other studies that have shown strong associations (3). This could be related to the higher prevalence of smoking in the youngest age period compared to the last examination, representing the older period. More years of education predicted more stature shrinkage in the latest observation period only, which may be related to societal trends towards longer educations for women. Height shrinkage is associated with poor health (1,4,7,8). Vertebral discs degenerate far earlier than other musculoskeletal tissues (1) and can lead to back pain and disability in the older adults (1) and in severe cases even vertebral fractures, which have been shown to have direct or indirect consequences such as decreased mobility, depression, hospitalization, and mortality (21). Height loss exceeding 4 cm has been found to be related to fracture probability of over 50% (3).

Panoramic radiography indices, cortical thickness, erosion, and sparse trabeculation, have been suggested as screening tools for identifying risk groups with osteoporosis (15,16,23). Cortical erosion, assessed at dental practices, has been shown to be useful in the identification of undetected low BMD and spine fractures among postmenopausal women (16,17).

For peer review only

For peer review only - http://bmjopen.bmj.com/site/about/guidelines.xhtml

However, Okabe et al. 2008 found no significant correlations between cortical measurements and the occurrence of fractures in a group of 80-years old Japanese men and women (14). The difference in the results may be attributed to ethnicity-related anthropometric differences , the fact that Swedish and Norwegian women have the highest frequencies of fragility fractures in the world (26). Another difference is that Okabe et al. studied five years of fracture incidence whereas Jonasson et al investigated the same over ten years.

Mandibular cortical width is associated with BMD (15,19,30). A previous study in this cohort of Swedish women at ages 62 to 78 years investigated mandibular cortical erosion and thickness as predictors of future osteoporotic fractures and found that severely eroded cortices predicted fractures, but cortical width did not (27). Sparse trabeculation was shown to be associated with a twofold higher risk for future fracture in a younger group and with a three- to fourfold higher risk for future fracture in an older group (28). As far as we know, relationships between height loss and oral indices have not been examined before.

All bone structures undergo an alteration in the bone remodeling mechanism in connection with menopause, leading to greater bone loss than bone formation (31). The imbalance results in trabecular thinning, increase in intra-trabecular spacing, loss of connectivity in the meshwork and intra-cortical erosion, thereby leading to diminished bone strength and increased risk of fracture, observed with increasing age (32,33). In long bones, a thin cortex and porosity, along with the body height have been shown to be associated with fracture risk (34). In that study, taller women had thinner cortical bone (in the appendicular skeleton) and therefore had a greater risk for fractures. This, in general, could also be applied to the pathophysiology behind cortical erosion in the vertebra (34).

A study has described the differences between mandibular versus long bones in terms of osteogenic potential and bone homeostasis (35). Despite the differences in embryological

For peer review only - http://bmjopen.bmj.com/site/about/guidelines.xhtml

origin, menopause related cortical thinning should be an expected finding in the jawbones as well (35). An experimental study examined differences in the osteoclastic process at different sites of the body. Results showed no significant difference in terms of resorption rate between long bones and jawbones, although larger (yet fewer) osteoclasts were detected in the jawbones (36).

The vertebral body volume is largely trabecular with only a thin shell of cortical bone. The trabecular bone is remodeled and lost more rapidly than the cortical bone, but later in life, the main bone loss occurs in the cortical compartment (34). With aging, progressive bone loss leads to less dense bone pattern. Therefore, the axial compressive load on the vertebra is distributed on fewer and thinner trabeculae, resulting in an increasing risk for fractures (37,38).

In our study population, the sparse trabeculation and the cortical erosion of the mandible could be seen to mimic the status of the vertebral trabeculae and intracortical erosion of the thin cortical shell of the vertebrae, which would, in turn, explain the potential height reduction. These two bone-related risk factors are important for the development of height loss as well as osteoporosis. A vertebral fracture is often asymptomatic, but it should not be ignored since it is a highly significant risk factor for future fractures, and if symptomatic, it is difficult to relieve the patients suffering.

Visual assessments of the trabecular and cortical bone as conducted in this study are not routine in dental practice today, but simplified screening procedures using artificial intelligence may change this in the future (39). Bone turnover is probably the highest in the mandibular alveolar process (40), and therefore a collaboration with physicians may open opportunities for risk detection, assessment and preventive actions addressing osteoporosis,

For peer review only - http://bmjopen.bmj.com/site/about/guidelines.xhtml

and pathological fractures. Cortical erosion is easily evaluated using panoramic radiographs, whereas the trabeculation is best assessed on periapical radiographs.

In conclusion, mandibular bone structure alterations such as severe cortical erosion and sparse trabeculation predicted stature shrinkage. They may therefore serve as important proxy indicators when screening in the early phases of bone degenerative pathogenesis, signaling the ongoing bone remodeling and height loss and the need for further clinical attention to the older women at risk for stature shrinkage.

Contributorship statement: NNG and LL initiated the study. VS and GJ did the acquisition of data. All authors contributed to the analysis and interpretation of data. VS and NNG did the statistical analyses. NNG wrote the first draft of the manuscript. All authors critically revised the manuscript and approved the final version. LL obtained the funding.

Competing interest: None

Funding: This work was supported by grants from the Swedish Research Council for Health, Wing Life and Welfare (Forte, EpiLife 200i6-1506), and the Swedish ALF-agreement (ALFGBG 720201).

Data sharing statement: No additional data available.

Ethics Approval: The Prospective Population Study of Women in Gothenburg has been approved by the Regional Ethics Review Board of the University of Gothenburg (*DNR 65-80, 179-92, T453-04*). The examinations comply with the Declaration of Helsinki and informed consent has been obtained from the subjects.

For peer review only - http://bmjopen.bmj.com/site/about/guidelines.xhtml

References

1. Hillier TA, Lui LY, Kado DM, LeBlanc ES, Vesco KK, Bauer DC, et al. Height loss in older women: risk of hip fracture and mortality independent of vertebral fractures. *J Bone Miner Res*. 2012;27:153-159. doi: 10.1002/jbmr.558
2. Gavriilidou NN, Pihlsgard M, Elmstahl S. Anthropometric reference data for elderly Swedes and its disease-related pattern. *Eur J Clin Nutr.* 2015;69:1066-1075. doi: 10.1038/ejcn.2015.73.
3. Pluskiewicz W, Adamczyk P, Drozdzowska B. The significance of height loss in postmenopausal women. The results from GO Study. *Int J Clin Pract.* 2021;75:e14009. doi: 10.1111/ijcp.14009.
4. Klingberg S, Mehlig K, Dangol R, Björkelund C, Heitmann BL, Lissner L. Loss of height predicts total and cardiovascular mortality: a cohort study of northern European women. *BMJ Open.* 2021;11:e049122. doi: 10.1136/bmjopen-2021-049122.
5. Perissinotto E, Pisent C, Sergi G, Grigoletto F, Group IW. Anthropometric measurements in the elderly: age and gender differences. *Br J Nutr.* 2002;87:177-186. doi: 10.1079/bjn2001487.
6. Gavriilidou NN, Pihlsgard M, Elmstahl S. High degree of BMI misclassification of malnutrition among Swedish elderly population: Age-adjusted height estimation using knee height and demispan. *Eur J Clin Nutr.* 2015;69:565-571. doi: 10.1038/ejcn.2014.183.
7. Urban JP, Roberts S. Degeneration of the intervertebral disc. *Arthritis Res Ther.* 2003;5:120-130. doi: 10.1186/ar629.
8. Wannamethee SG, Shaper AG, Lennon L, Whincup PH. Height loss in older men: associations with total mortality and incidence of cardiovascular disease. *Arch Intern Med.* 2006;166:2546-2552. doi: 10.1001/archinte.166.22.2546.

For peer review only - http://bmjopen.bmj.com/site/about/guidelines.xhtml

9. Huang W, Lei X, Ridder G, Strauss J, Zhao Y. Health, Height, Height Shrinkage, and SES at Older Ages: Evidence from China. *Am Econ J Appl Econ.* 2013;5:86-121. doi: 10.1257/app.5.2.86

10. Kanis JA. Assessment of fracture risk and its application to screening for postmenopausal osteoporosis: synopsis of a WHO report. WHO Study Group. *Osteoporos Int*. 1994;4:368-381. doi: 10.1007/BF01622200

11. Jonasson G, Bankvall G, Kiliaridis S. Estimation of skeletal bone mineral density by means of the trabecular pattern of the alveolar bone, its interdental thickness, and the bone mass of the mandible. *Oral Surg Oral Med Oral Pathol Oral Radiol Endod.* 2001;92:346-352. doi: 10.1067/moe.2001.116494.

12. Lindh C, Horner K, Jonasson G, Olsson P, Rohlin M, Jacobs R, et al. The use of visual assessment of dental radiographs for identifying women at risk of having osteoporosis: the OSTEODENT project. *Oral Surg Oral Med Oral Pathol Oral Radiol Endod.* 2008;106:285-293. doi: 10.1016/j.tripleo.2007.09.008.

13. Lindh C, Petersson A, Rohlin M. Assessment of the trabecular pattern before endosseous implant treatment: diagnostic outcome of periapical radiography in the mandible. *Oral Surg Oral Med Oral Pathol Oral Radiol Endod*. 1996;82:335-243. doi: 10.1016/s1079-2104(96)80363-5

14. Okabe S, Morimoto Y, Ansai T, Yoshioka I, Tanaka T, Taguchi A, et al. Assessment of the relationship between the mandibular cortex on panoramic radiographs and the risk of bone fracture and vascular disease in 80-year-olds. *Oral Surg Oral Med Oral Pathol Oral Radiol Endod.* 2008;106:433-424. doi: 10.1016/j.tripleo.2007.09.013.

15. Taguchi A. Panoramic radiographs for identifying individuals with undetected osteoporosis. *Japanese Dental Science Review.* 2009;45:109-120. doi.org/10.1016/j.jdsr.2009.05.001

For peer review only - http://bmjopen.bmj.com/site/about/guidelines.xhtml

16. Taguchi A, Ohtsuka M, Nakamoto T, Naito K, Tsuda M, Kudo Y, et al. Identification of post-menopausal women at risk of osteoporosis by trained general dental practitioners using panoramic radiographs. *Dentomaxillofac Radiol*. 2007;36:149-154. doi: 10.1259/dmfr/31116116.

17. Lissner L, Skoog I, Andersson K, Beckman N, Sundh V, Waern M, et al. Participation bias in longitudinal studies: experience from the Population Study of Women in Gothenburg, Sweden. *Scand J Prim Health Care.* 2003;21:242-247. doi: 10.1080/02813430310003309-1693.

18. Bengtsson C, Ahlqwist M, Andersson K, Bjorkelund C, Lissner L, Soderstrom M. The Prospective Population Study of Women in Gothenburg, Sweden, 1968-69 to 1992-93. A 24-year follow-up study with special reference to participation, representativeness, and mortality. *Scand J Prim Health Care.* 1997;15:214-219. doi: 10.3109/02813439709035031.

19. Klemetti E, Kolmakov S, Kroger H. Pantomography in assessment of the osteoporosis risk group. *Scand J Dent Res*. 1994;102:68-72. doi: 10.1111/j.1600-0722.1994.tb01156.x.

20. Sagiv M, Vogelaere PP, Soudry M, Ehrsam R. Role of physical activity training in attenuation of height loss through aging. *Gerontology*. 2000;46:266-270. doi: 10.1159/000022170.

21. Xu W, Perera S, Medich D, Fiorito G, Wagner J, Berger LK, et al. Height loss, vertebral fractures, and the misclassification of osteoporosis. *Bone.* 2011;48:307-311. doi: 10.1016/j.bone.2010.09.027.

22. Thornton MJ, Sedlak CA, Doheny MO. Height change and bone mineral density: revisited. *Orthop Nurs.* 2004;23:315-320. doi: 10.1097/00006416-200409000-00007.

For peer review only

23. Jonasson G, Sundh V, Ahlqwist M, Hakeberg M, Bjorkelund C, Lissner L. A prospective study of mandibular trabecular bone to predict fracture incidence in women: a low-cost screening tool in the dental clinic. *Bone.* 2011;49:873-879. doi: 10.1016/j.bone.2011.06.036.

24. Grimby G, Börjesson M, Jonsdottir IH, Schnohr P, Thelle DS, Saltin B. The "Saltin-Grimby Physical Activity Level Scale" and its application to health research. *Scand J Med Sci Sports.* 2015;25 Suppl 4:119-25. doi: 10.1111/sms.12611

25. Kaufman JS, Cooper RS. Seeking Causal Explanations in Social Epidemiology. *Am J Epidemiol.* 1999;150:113-120. doi: 10.1093/oxfordjournals.aje.a009969.

26. SBU. Osteoporos – prevention, diagnostik och behandling. En systematisk litteraturöversikt. Stockholm.: Statens beredning för medicinsk utvärdering (SBU). 2003.

27. Jonasson GB, Sundh V, Hakeberg M, Ahlqwist M, Lissner L, Hange D. Evaluation of clinical and radiographic indices as predictors of osteoporotic fractures: a 10-year longitudinal study. *Oral Surg Oral Med Oral Pathol Oral Radiol.* 2018;125:487-494. doi: 10.1016/j.oooo.2017.11.009.

28. Sundh V, Hange D, Ahlqwist M, Hakeberg M, Lissner L, Jonasson G. FRAX and mandibular sparse trabeculation as fracture predictors: a longitudinal study from 1980 to 2002. *Eur J Oral Sci.* 2017;125:135-140. doi: 10.1111/eos.12341.

29. Mai X, Marshall B, Hovey KM, Sperrazza J, Wactawski-Wende J. Risk factors for 5-year prospective height loss among postmenopausal women. *Menopause.* 2018;25:883-889. doi: 10.1097/GME.0000000000001108.

30. Devlin H, Horner K. Mandibular radiomorphometric indices in the diagnosis of reduced skeletal bone mineral density. *Osteoporos Int.* 2002;13:373-378. doi: 10.1007/s001980200042.

For peer review only

For peer review only - http://bmjopen.bmj.com/site/about/guidelines.xhtml

31. Lerner UH. Bone remodeling in post-menopausal osteoporosis. *J Dent Res.* 2006;85:584-595. doi: 10.1177/154405910608500703.

32. Bala Y, Zebaze R, Seeman E. Role of cortical bone in bone fragility. *Curr Opin Rheumatol.* 2015;27(4):406-13. doi: 10.1097/BOR.0000000000000183.

33. Bjornerem A, Wang X, Bui M, Ghasem-Zadeh A, Hopper JL, Zebaze R, et al. Menopause-Related Appendicular Bone Loss is Mainly Cortical and Results in Increased Cortical Porosity. *J Bone Miner Res.* 2018;33:598-605. doi: 10.1002/jbmr.3333.

34. Bjornerem A, Bui QM, Ghasem-Zadeh A, Hopper JL, Zebaze R, Seeman E. Fracture risk and height: an association partly accounted for by cortical porosity of relatively thinner cortices. *J Bone Miner Res.* 2013;28:2017-2026. doi: 10.1002/jbmr.1934.

35. Aghaloo TL, Chaichanasakul T, Bezouglaia O, Kang B, Franco R, Dry SM, et al. Osteogenic potential of mandibular vs. long-bone marrow stromal cells. *J Dent Res* 2010;89:1293-1298. doi: 10.1177/0022034510378427..

36. de Souza Faloni AP, Schoenmaker T, Azari A, Katchburian E, Cerri PS, de Vries TJ, et al. Jaw and long bone marrows have a different osteoclastogenic potential. *Calcif Tissue Int.* 2011;88:63-74. doi: 10.1007/s00223-010-9418-4.

37. Adams MA, Hutton WC. The Mechanical Function of the Lumbar Apophyseal Joints. *Spine.* 1983;8. doi: 10.1097/00007632-198304000-00017.

38. Giambini H, Salman Roghani R, Thoreson AR, Melton LJ, 3rd, An K-N, Gay RE. Lumbar trabecular bone mineral density distribution in patients with and without vertebral fractures: a case-control study. *Eur Spine J.* 2014;23:1346-1353. doi: 10.1007/s00586-014-3205-2.

39. Jonasson G, Billhult A. Mandibular bone structure, bone mineral density, and clinical variables as fracture predictors: a 15-year follow-up of female patients in a dental

For peer review only - http://bmjopen.bmj.com/site/about/guidelines.xhtml

clinic. *Oral Surg Oral Med Oral Pathol Oral Radiol.* 2013;116:362-368. doi: 10.1016/j.oooo.2013.06.009.

40. Jonasson G, Skoglund I, Rythén M. The rise and fall of the alveolar process: Dependency of teeth and metabolic aspects. *Arch Oral Biol*. 2018;96:195-200. https://doi.org/10.1016/j.archoralbio.2018.09.016

For peer review only

Figure legend

Figure 1: Flow chart.

For peer review only

For peer review only - http://bmjopen.bmj.com/site/about/guidelines.xhtml

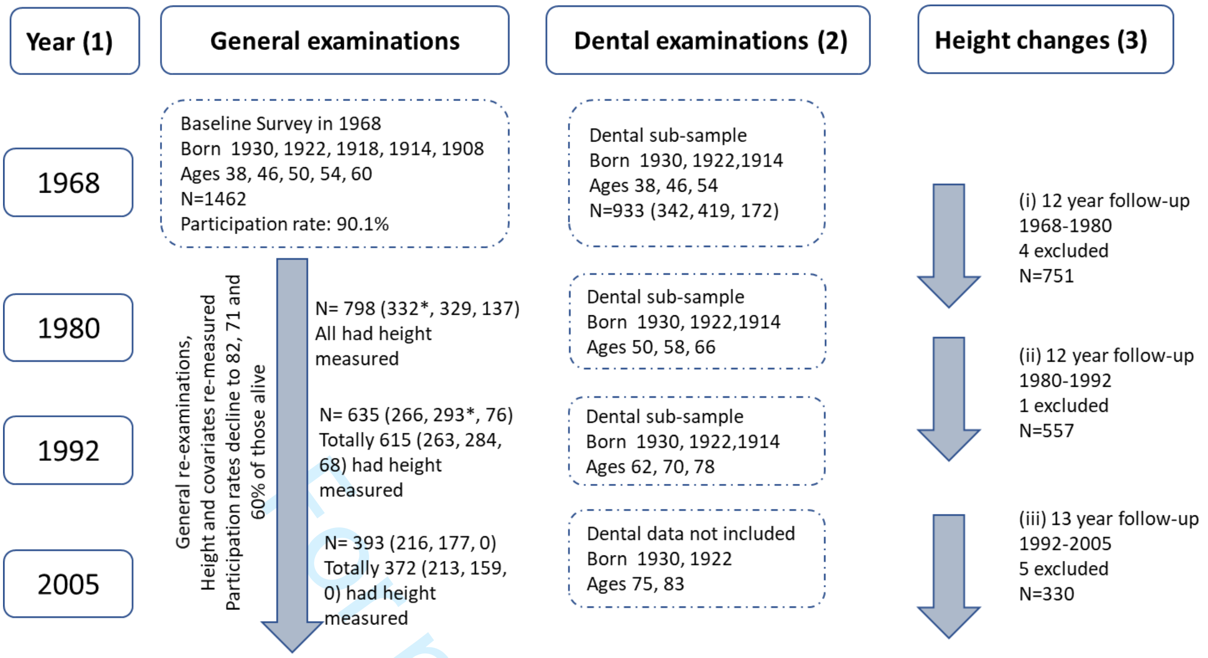

(1) Each examination period starting in September and ending in June of following year, i.e., 1968-69, 1980-81, 1992-93, 2005-06
(2) Dental examination conducted shortly after general examination in three of original age groups. Mandibular bone structure (cortical and trabecular bone) is the main independent variable
(3) Heights were measured at general examinations, and the change in height was estimated at 12- or 13-year intervals, after excluding any increases of over 2 cm

For peer review only

STROBE 2007 (v4) Statement—Checklist of items that should be included in reports of *cohort studies*

Section/Topic	Item #	Recommendation	Reported on page #
**Title and abstract**	1	(*a*) Indicate the study's design with a commonly used term in the title or the abstract	1 and 2
		(*b*) Provide in the abstract an informative and balanced summary of what was done and what was found	2
**Introduction**			
Background/rationale	2	Explain the scientific background and rationale for the investigation being reported	5
Objectives	3	State specific objectives, including any prespecified hypotheses	6
**Methods**			
Study design	4	Present key elements of study design early in the paper	2-5
Setting	5	Describe the setting, locations, and relevant dates, including periods of recruitment, exposure, follow-up, and data collection	2, 5
Participants	6	(*a*) Give the eligibility criteria, and the sources and methods of selection of participants. Describe methods of follow-up	5
		(*b*) For matched studies, give matching criteria and number of exposed and unexposed	-
Variables	7	Clearly define all outcomes, exposures, predictors, potential confounders, and effect modifiers. Give diagnostic criteria, if applicable	7, 8
Data sources/ measurement	8*	For each variable of interest, give sources of data and details of methods of assessment (measurement). Describe comparability of assessment methods if there is more than one group	*7, 8*
Bias	9	Describe any efforts to address potential sources of bias	11, 12
Study size	10	Explain how the study size was arrived at	7
Quantitative variables	11	Explain how quantitative variables were handled in the analyses. If applicable, describe which groupings were chosen and why	8
Statistical methods	12	(*a*) Describe all statistical methods, including those used to control for confounding	8
		(*b*) Describe any methods used to examine subgroups and interactions	8, 10
		(*c*) Explain how missing data were addressed	6
		(*d*) If applicable, explain how loss to follow-up was addressed	-
		(*e*) Describe any sensitivity analyses	-
**Results**			

For peer review only

Participants	13*	(a) Report numbers of individuals at each stage of study—eg numbers potentially eligible, examined for eligibility, confirmed eligible, included in the study, completing follow-up, and analysed	6, 7 +fig 1
		(b) Give reasons for non-participation at each stage	6, 7
		(c) Consider use of a flow diagram	Fig 1
Descriptive data	14*	(a) Give characteristics of study participants (eg demographic, clinical, social) and information on exposures and potential confounders	6, 7
		(b) Indicate number of participants with missing data for each variable of interest	Table 1
		(c) Summarise follow-up time (eg, average and total amount)	Fig 1 + tables
Outcome data	15*	Report numbers of outcome events or summary measures over time	5-8
Main results	16	(*a*) Give unadjusted estimates and, if applicable, confounder-adjusted estimates and their precision (eg, 95% confidence interval). Make clear which confounders were adjusted for and why they were included	Tables
		(*b*) Report category boundaries when continuous variables were categorized	Tables
		(*c*) If relevant, consider translating estimates of relative risk into absolute risk for a meaningful time period	-
Other analyses	17	Report other analyses done—eg analyses of subgroups and interactions, and sensitivity analyses	-
**Discussion**			
Key results	18	Summarise key results with reference to study objectives	10
**Limitations**			
Interpretation	20	Give a cautious overall interpretation of results considering objectives, limitations, multiplicity of analyses, results from similar studies, and other relevant evidence	14
Generalisability	21	Discuss the generalisability (external validity) of the study results	2, 11
**Other information**			
Funding	22	Give the source of funding and the role of the funders for the present study and, if applicable, for the original study on which the present article is based	15

*Give information separately for cases and controls in case-control studies and, if applicable, for exposed and unexposed groups in cohort and cross-sectional studies.

Note: An Explanation and Elaboration article discusses each checklist item and gives methodological background and published examples of transparent reporting. The STROBE checklist is best used in conjunction with this article (freely available on the Web sites of PLoS Medicine at http://www.plosmedicine.org/, Annals of Internal Medicine at http://www.annals.org/, and Epidemiology at http://www.epidem.com/). Information on the STROBE Initiative is available at www.strobe-statement.org.

For peer review only

BMJ Open

Does mandibular bone structure predict subsequent height loss? A longitudinal cohort study of women in Gothenburg, Sweden

Journal:	*BMJ Open*
Manuscript ID	bmjopen-2022-066844.R1
Article Type:	Original research
Date Submitted by the Author:	10-Nov-2022
Complete List of Authors:	Gavriilidou, Nivetha N; Karolinska Institute, Department of Dental Medicine<br>Jonasson, Grethe; University of Gothenburg, Department of Behavioral and Community Dentistry<br>Sundh, Valter; University of Gothenburg, School of Public Health and Community Medicine, Institute of Medicine, Sahlgrenska Academy,<br>Rothenberg, Elisabet ; Kristianstad University, Department of Nursing and Integrated Health Sciences<br>Lissner, Lauren; University of Gothenburg, School of Public Health and Community Medicine, Institute of Medicine, Sahlgrenska Academy,
**Primary Subject Heading**:	Dentistry and oral medicine
Secondary Subject Heading:	Dentistry and oral medicine
Keywords:	ORAL MEDICINE, Bone diseases < ORTHOPAEDIC & TRAUMA SURGERY, PUBLIC HEALTH

SCHOLARONE™
Manuscripts

BMJ

I, the Submitting Author has the right to grant and does grant on behalf of all authors of the Work (as defined in the below author licence), an exclusive licence and/or a non-exclusive licence for contributions from authors who are: i) UK Crown employees; ii) where BMJ has agreed a CC-BY licence shall apply, and/or iii) in accordance with the terms applicable for US Federal Government officers or employees acting as part of their official duties; on a worldwide, perpetual, irrevocable, royalty-free basis to BMJ Publishing Group Ltd ("BMJ") its licensees and where the relevant Journal is co-owned by BMJ to the co-owners of the Journal, to publish the Work in this journal and any other BMJ products and to exploit all rights, as set out in our licence.

The Submitting Author accepts and understands that any supply made under these terms is made by BMJ to the Submitting Author unless you are acting as an employee on behalf of your employer or a postgraduate student of an affiliated institution which is paying any applicable article publishing charge ("APC") for Open Access articles. Where the Submitting Author wishes to make the Work available on an Open Access basis (and intends to pay the relevant APC), the terms of reuse of such Open Access shall be governed by a Creative Commons licence – details of these licences and which Creative Commons licence will apply to this Work are set out in our licence referred to above.

Other than as permitted in any relevant BMJ Author's Self Archiving Policies, I confirm this Work has not been accepted for publication elsewhere, is not being considered for publication elsewhere and does not duplicate material already published. I confirm all authors consent to publication of this Work and authorise the granting of this licence.

For peer review only

Does mandibular bone structure predict subsequent height loss? A longitudinal cohort study of women in Gothenburg, Sweden

Nivetha N Gavriilidou DDS MPH PhD, [a] Grethe Jonasson DDS PhD, [b] Valter Sundh BSc, [c] Elisabet Rothenberg PhD, [d] Lauren Lissner MPH PhD. [e]

[a] Karolinska Institute, Department of Dental Medicine, Institute of Odontology, Karolinska Institute, Sweden. Email: nivetha.gavriilidou@ki.se

[b] Department of Behavioral and Community Dentistry, Institute of Odontology at the Sahlgrenska Academy, University of Gothenburg, Sweden. Email: grethe.jonasson@gu.se

[c.] School of Public Health and Community Medicine, Institute of Medicine, Sahlgrenska Academy, University of Gothenburg, Gothenburg, Sweden. Email: valter.sundh@gu.se

[d] Department of Nursing and Integrated Health Sciences, Faculty of Health Sciences, Kristianstad University, Kristianstad, Sweden. Email: elisabet.rothenberg@hkr.se

[e] School of Public Health and Community Medicine, Institute of Medicine, Sahlgrenska Academy, University of Gothenburg, Sweden. Email: lauren.lissner@gu.se

Corresponding author: Grethe Jonasson, Department of Behavioral and Community Dentistry, Institute of Odontology at the Sahlgrenska Academy, University of Gothenburg, Sweden. Email: grethe.jonasson@gu.se; tel +46 709285671

Short title: Mandibular bone structure and stature shrinkage.

For peer review only - http://bmjopen.bmj.com/site/about/guidelines.xhtml

ABSTRACT

Background Several risk factors for loss of height with increasing age have been identified.

Objective To investigate if mandibular bone structure predicts future height loss in middle-aged and elderly Swedish women.

Design Prospective cohort study with longitudinally measured heights, radiographical assessments of the cortical bone using Klemetti´s Index (normal, moderate, or severe eroded cortex), and classification of the trabecular bone using an index proposed by Lindh et al. (sparse, mixed, or dense trabeculation). No intervention was performed.

Setting Gothenburg, Sweden.

Participants A population-based sample of 937 Swedish women born in 1914, 1922 and 1930 was recruited. At the baseline examination, the ages were 38, 46, and 54. All had undergone a dental examination with panoramic radiographs of the mandible, and a general examination including height measurements on at least 2 occasions.

Main outcome measure Height loss was calculated over three 12-13-year periods (1968-80, 1980-92, 1992- 2005).

Main results. Mean annual height loss measures were 0.075cm/year, 0.08 cm/year, and 0.18 cm/year over the 3 observation intervals, corresponding to absolute decreases of 0.9 cm, 1.0 cm, and 2.4 cm. Cortical erosion in 1968 and 1980 significantly predicted height loss 12 years later. Sparse trabeculation in 1968, 1980 and 1992 also predicted significant shrinkage over 12 or 13 years. Multivariable regression analyses adjusting for baseline covariates height, birth year, physical activity, smoking, body mass index, and education yielded consistent findings.

For peer review only

For peer review only - http://bmjopen.bmj.com/site/about/guidelines.xhtml

Conclusion Mandibular bone structure characteristics such as severe cortical erosion and sparse trabeculation may serve as early risk factors for height loss. Since most individuals visit their dentist at least every two years and radiographs are taken, a collaboration between dentists and physicians may open opportunities for predicting future risk of height loss.

Keywords Ageing, body height, cortical bone, dental radiography, mandible, stature decline, trabecular bone, women's health.

For peer review only

Strengths and limitations of this study

- The strengths are the prospective design, the population-based sample, the long follow-up, repeated measurements of height for the same individuals, and that our findings can be applicable to many settings.
- The main limitation is that loss of height could reflect different conditions which may make accurate height estimates difficult.
- The participation rates became lower in the later years, and the drop out for different reasons, mortality included, probably differs between age groups, which might impact the mean height losses.

For peer review only

For peer review only - http://bmjopen.bmj.com/site/about/guidelines.xhtml

INTRODUCTION

Height loss in older women is incrementally more pronounced at ages over 75 years. It has been found to be associated with increased morbidity and mortality (1-4). Several causes have been identified that lead to loss of height among older adults. Height loss could indicate age-associated progressive skeletal deformation such as kyphoscoliosis flattening of plantar arch and altered posture (5,6), and/or degenerative processes including osteoporosis, vertebral compression and attrition of intervertebral discs and vertebral fractures (7). These alterations make accurate height estimation a challenge. Comorbidities of major height loss in older adults include functional impairments such as dependence in daily life activities, cognitive impairment, in comparison with their healthy counterparts, and increased overall mortality risk (1,3). Height loss over 5cm has been shown to predict non-spinal fractures such as hip fractures and mortality independent of vertebral fractures (1-3, 5-9). Height loss in women exceeding 4 cm has been found to be related to fracture probability of over 50% (3). In men, health consequences of height loss over 3 cm include increased risk of all-cause mortality and coronary heart disease (8).

Osteoporosis is a widely acknowledged risk factor for future fractures in the elderly. According to the World Health Organization, it is defined as bone density lower than 2.5 standard deviations under that of young women using the bone mineral density of the hip with dual X-ray absorptiometry (DXA) (10). Simple, practical, and relevant techniques are needed for early identification of the onset or extent of skeletal degeneration. Therefore, researchers have used oral variables such as optical density (11), sparse trabeculation (11-13), cortical width (14), and cortical erosion (14-16) for the prediction of osteoporosis and fracture.

Intraoral radiographs are usually an integral part of the routine oral examination. Assessment of mandibular structure, the cortical and trabecular bone pattern may be studied through visual inspection, hence providing a simple screening method for the evaluation of

For peer review only - http://bmjopen.bmj.com/site/about/guidelines.xhtml

the bone condition. The hypothesis of the present study is that the mandibular bone contains sufficient information about the general bone condition to predict future height loss. The objective is to investigate two proxies for general bone mineral density, namely mandibular cortical erosion, and trabecular coarseness, as predictors of height loss over subsequent periods of 12-13 years.

METHODS

Study population

The Prospective Population Study of Women in Gothenburg, Sweden, is an ongoing longitudinal study initiated in 1968 in a cohort of women aged 38, 46, 50, 54, or 60 years. At that time, a representative sample of women in Gothenburg was identified using the Revenue Office register with respect to the dates of birth. From the full baseline cohort, those born in 1914, 1922, and 1930 were included in the present analytic sample (flowchart, Figure 1). The original survey included general health and dental examinations, which were repeated over the years. Each wave of survey was started in the fall and completed in the spring of the following year, i.e., 1968-69, 1980-81, 1992-93, and 2004-05, and is hereafter referred to by its starting year. The participation rate in the initial examination was over 90% (17,18). A non-participation analysis of the 24-year follow-up in 1992 showed that the women who declined to participate in the first survey did not differ significantly from participants except for the long-term survival which was lower in the initial refusers. The sample remained representative of the general population in terms of parameters such as blood pressure, weight, height, BMI, waist and hip circumference, serum cholesterol, triglyceride, and urate (18).

Inclusion criteria

For peer review only - http://bmjopen.bmj.com/site/about/guidelines.xhtml

In total, 937 women born in 1914, 1922 or 1930, were included in the present analysis. The inclusion criteria were availability of at least one panoramic radiograph in 1968, 1980, or 1992 from which it was possible to assess the mandibular cortex erosion and sparsity of trabeculae, plus availability of a baseline and at least one subsequent height measure. In the first period (1968-1989), four individuals were excluded due to an *increase* in height of at least 2 cm. Number excluded 1980-1992 was one, and it was five in the period 1992-2005. Results from previous observational studies using the same cohorts have shown that the number of participants should be sufficient for studying common health endpoints.

Patient and Public Involvement

No patients involved.

Main outcome

The main outcome variable was height loss during the 12-13 years following the dental examination. At all examinations, height and weight were assessed by standardized anthropometric measurement techniques. A stadiometer was used to measure the participants' body heights (without shoes). Measurement of height was generally performed early in the day. Height loss was calculated as baseline height minus height at follow-up. Thus, a positive value indicated height loss.

Height change was assessed from follow-up examinations 12-13 years later, until 2005. The exclusion criterion was an observed *increase* in height of at least 2 cm during any of these follow-up periods, attributable to probable measurement errors.

There were three 12–13-year follow-up periods namely 1968-1980, 1980-1992, and 1992-2005, where the first period comprised middle-aged women initially aged 38, 46, 54 years, the second period with a subset of these women from ages 62, 70 and 78 years, and the third and the latest period starting at ages 75 and 83 years (no follow-up from 1914 birth cohort) (Figure 1).

For peer review only - http://bmjopen.bmj.com/site/about/guidelines.xhtml

Bone structure and other covariates

Radiographic assessment of mandibular bone structures was included as part of the dental examination. In the present analysis, the main independent variables were two components of mandibular bone status. On visual inspection of the panoramic radiograph of a mandible, the cortical bone was categorized using Klemetti´s Index as normal, moderate, or severe eroded cortex (19). A normal cortex means an even endosteal margin; a moderate eroded cortex has semilunar defects in the endosteal margin, whereas a severe eroded cortex has heavy endosteal cortical porosities. The second component, the trabeculation, was classified using an index proposed by Lindh et al. 1996 as sparse, mixed, or dense (12,13). Sparse trabeculation means large intertrabecular spaces in the premolar area. Dense trabeculation has small intertrabecular spaces. Mixed dense and sparse trabeculation is mostly dense at the crest and sparse apically.

Baseline variables such as weight, height, body mass index (BMI), smoking, physical activity, and education, were considered potential covariates that could be associated with mandibular erosion and trabecular pattern, as well as height loss (20-23). The survey questionnaire regarding smoking habit had 6 possible responses: Never smoked (0), Previous smoker but quit in the last 15 years (1), Previous smoker but quit since last year (2), Quit smoking since last year (3), Current smoker without deep inhalation of the smoke (4), and Current smoker with deep inhalation of the smoke (5). This variable was dichotomized to "*No smoking*" (0) including response options 0, 1, 2, and "*Smoking*" (1) including options 3, 4, or 5. The variable physical activity was categorized as (i) almost no physical activity or none (sedentary), (ii) at least 4 hours/ a week of physical activity, (iii) regular physical activity and (iiii) regular intense training and competitions (24). This variable was dichotomized as: "*physically inactive*" (0) including the first response and "*physically active*" (1) including the latter three choices. Education was considered a proxy for socioeconomic status as proposed

For peer review only - http://bmjopen.bmj.com/site/about/guidelines.xhtml

by Kaufmann (25) and categorized as low: pre-high school (up to 9 years); medium: high school with ≤2 years at university; and high: 3 or more years at university.

Statistical analyses

The mandibular bone variables are categorical. The Kruskal-Wallis test was used to analyze differences in height loss between the three categories of cortical erosion, whereas Mann-Whitney Two-Sample Test was used for differences between two trabeculation groups. In the analyses, the trabeculation variable was dichotomized into sparse and normal (mixed and dense). Height, height loss and BMI were continuous variables, whereas smoking and physical activity were dichotomized as described above; education was also a categorical variable with three categories. In order to test the importance of lifestyle factors, multiple regression analyses were performed, where height loss was the dependent variable. The following variables were independent covariates: the two mandibular bone variables, smoking, physical activity, height, body mass index (BMI), education, and age (birth year). BMI was considered as a measure of possible underweight/normal weight/overweight and therefore included.

The significance level was p<0.05). Statistical analyses were performed with SAS version 9.2 software (SAS Institute Inc., Cary, NC).

RESULTS

Selected characteristics of the sample are shown in Table 1. Mean age at the four height measurements (year 1968, 1980, 1992, and 2005) were 44.9, 56.9, 68.0, and 78.8 years respectively, corresponding to three periods of height change (1968-80, 1980-92, and 1992-2005). There was a rising trend in the proportion of women with severe cortical erosion from 3.2% among the participants in the period 1968-1980, to 11.1% in the period 1980-1992 and

For peer review only - http://bmjopen.bmj.com/site/about/guidelines.xhtml

up to 49.8% in the period 1992-2005. Similarly, prevalence of sparse trabeculation increased from 21.8% among the participants 1968-1980, to 37.2% in 1980-1992 and up to 48.3% in the period 1992-2005. At the same time, the mean height loss over the three survey periods was 0.9 cm, 1.0 cm, and 2.4 cm, with corresponding annual decreases of 0.075, 0.08, and 0.18 cm/year (Table 1).

Table 1: Descriptive characteristics of the population sub-samples born in 1914, 1922 and 1930 at examination year 1968 followed up across periods 1968-80, 1980-92, 1992-2005.

	**1968**	**1980**	**1992**	**2005**
Age of women born 1930 years (N)	38 (342)	50 (289)	62 (251)	75 (190)
Born 1922	46 (419)	58 (327)	70 (239)	83 (140)
Born 1914	54 (172)	66 (135)	78 (67)	91 (0) [a]
	Total (933)	Total (751)	Total (557)	Total (330)
**Parameters at the end of the period**	**1968**	**1968-80**	**1980-92**	**1992-2005**
Mean age (SD)	44.9 (5.8)	56.9 (5.8)	68.0 (5.4)	78.8 (3.8)
Mean height in cm (SD)	163.9 (6.0)	163.0 (6.1)	162.1 (6.2)	160.4 (6.1)
Almost no physical activity N (%)	157 (16.8)	221 (29.4)	117 (21.0)	25 (9.3)
Active Smokers N (%)	308 (41.0)	257 (34.3)	115 (20.6)	30 (9.2)
Education level [b]				
Low	630 (67.7)	506 (67.4)	364 (65.4)	209 (63.3)
Medium	251 (27.0)	205 (27.3)	159 (28.5)	93 (28.2)
High	49 (5.3)	39 (5.2)	33 (5.9)	28 (8.5)
Mean body mass index (kg/m^2)	23.7 (3.7)	24.7 (3.9)	26.4 (4.2)	26.2 (4.5)
Weight (kg)	63.6 (10.8)	66.0 (11.2)	69.0 (11.9)	68.8 (11.8)
**Period 1968- 80**				
Mean height Loss (cm (SD))		0.90 (0.89)		
Mean annual height loss (cm/year)		0.075		
**Period 1980- 92**				
Mean height Loss (cm (SD))			1.0 (1.21)	
Mean annual height loss (cm/year)			0.08	
**Period 1992- 2005**				
Mean height Loss (cm (SD))				2.4 (1.54)
Mean annual height loss (cm/year)				0.18
**Mandibular parameters at the start of the period**				
Cortical Erosion *N (%)*				
Mild/None	583 (65.2)	451 (62.9)	175 (33.6)	5 (8.2)
Moderate	283 (31.7)	243 (33.9)	288 (55.3)	128 (42,0)
Severe erosion	28 (3.1)	23 (3.2)	58 (11.1)	152(49.8)
Trabeculation *N (%)*				
Normal	650 (79.4)	516 (78.2)	307 (62.8)	154 (51.7)
Sparse	169 (20.6)	144 (21.8)	182 (37.2)	144 (48.3)

Notes: Cortical bone was categorized using Klemetti´s Index as normal, moderate, or severe eroded cortex and the trabeculation classified into sparse or normal (mixed, or dense). Number excluded based on shrinkage values over period 1968-1980: 4, 1980-1992: 1, 1968-1992: 4, 1992-2005: 5.

For peer review only

[a]): There were none in this age group in the follow up period 1992-2005.
Not all participants with height measurements had an assessment of cortical erosion and even fewer had trabecular assessment due to edentulism.
[b]): Education level categories: Low: pre-high school level (up to 9 years), Medium: high school level with ≤2 years at university, High: 3 or more years at university.

Table 2 describes prospective analyses of how mandibular bone structure at the three age periods predicted height loss, without covariate adjustment.

Table 2: Height loss in relation to cortical erosion and trabecular spacing.

**Mandibular bone structure**	**Height loss (1968-80) [a]** **N= 751**			**Height loss (1980-92) [a)]** **N= 557**			**Height loss (1992-2005) [b)]** **N= 330**		
**Cortical Erosion**	Mean±SD	Rate/ year	p	Mean±SD	Rate/ year	p	Mean±SD	Rate/ year	p
Mild/None	0.84±0.84	0.07	<0.001	0.61±0.928	0.05	<0.001	2.48±1.42	0.19	ns
Moderate	0.99±0.92	0.08		1.04±1.17	0.09		2.21±1.22	0.17	
Severe	1.46±0.75	0.12		1.62±1.15	0.13		2.62±1.86	0.20	
**Trabecular bone**									
Normal	0.88±0.86	0.07	0.011	0.81±1.04	0.07	0.004	2.16±1.26	0.16	0.002
Sparse	1.01±0.99	0.08		1.11±1.15	0.09		2.72±1.82	0.20	

Notes: Bone assessments at the start year of 12-year periods (1968-1980, 1980-92 and 1992-2005). Mandibular cortical bone was categorized using Klemetti´s Index as normal, moderate, or severe eroded cortex and the trabeculation classified into sparse or normal (mixed, or dense).
[a)] Mean height loss was estimated from 12- year follow-up period. [b)] Mean height loss was estimated from 13-year follow-up period.

Height loss was highest in the groups with severe cortical erosion in the two first periods, whereas the group with sparse trabeculation had the highest bone loss during all three observation periods. On average, the difference in shrinkage between the two groups with milder versus severe erosion was 0.62 cm in the early period, 0.99 cm in the middle period, and only 0.14 cm in the last period (Table 2).

The difference in height loss between the group with normal/mixed and that with sparse trabeculation was 0.13 cm in the early period, 0.30 cm in the middle period, and 0.56 cm in

For peer review only - http://bmjopen.bmj.com/site/about/guidelines.xhtml

the last period. In all the three periods, the sparse trabeculation group presented more height loss than that with normal or mixed trabeculation (Table 2).

Multivariable linear regression analyses in Table 3 confirmed the significant associations between the height loss and cortical erosion during the periods 1968- 80 and 1980-92, but not 1992-2005, after adjusting for covariates as height, age (birth year), physical activity, smoking, education, and BMI. The trabeculation showed a significant correlation with height loss in all three periods (Table 3).

Table 3: Multivariable regression models predicting height loss over time intervals 1968-1980, 1980-92 and 1992-2005. The prediction is based on cortical erosion or trabeculation at baseline (1968), after adjusting for covariates height, year of birth, smoking, physical activity, education, and body mass index.

	[a]**Height loss (1968-80) N= 751**		[a] **Height loss (1980-92) N= 557**		[b] **Height loss (1992-2005) N=330**	
	Coefficient (Std Error)	P value	Coefficient Std Error	P value	Coefficient Std Error	P value
**Cortical Erosion**	**0.14 (0.06)**	**0.013**	**0.30 (0,09)**	**<0.001**	**0.15 (0.15)**	**0.338**
Height at baseline	0.01 (0.01)	0.217	0.02 (0.01)	0.088	0.02 (0.02)	0.156
Birth year	-0.05 (0.01)	<0.001	-0.06 (0.01)	<0.001	-0.02 (0.02)	0.321
Physical activity	-0.10 (0.07)	0.437	-0.25 (0.12)	0.036	0.10 (0.19)	0.481
Smoking	-0.04 (0.06)	0.517	0.06 (0.11)	0.579	0.34 (0.19)	0.013
Education	-0.01 (0.05)	0.938	0.09 (0.09)	0.299	0.39 (0.15)	0.009
BMI	0.01 (0.01)	0.194	-0.01 (0.02)	0.828	0.012 (0.03)	0.490
Unadjusted	0.21 (0.06)	<0.001	0.45 (0.08)	<0.001	0.18 (0.14)	0.195
**Trabeculation**	**0.17 (0.08)**	**0.030**	**0.24 (0.12)**	**0.021**	**0.40 (0.19)**	**0.032**
Height at baseline	0.01 (0.01)	0.048	0.02 (0.01)	0.015	0.02 (0.02)	0.359
Birth year	-0.06 (0.01)	<0.001	-0.06 (0.01)	<0.001	-0.01 (0.02)	0.554
Physical activity	-0.24 (0.07)	<0.001	-0.26 (0.12)	0.026	-0.01 (0.26)	0.977
Smoking	-0.04 (0.07)	0.576	-0.07 (0.10)	0.509	0.33 (0.19)	0.076
Education	-0.03 (0.06)	0.553	0.11 (0.08)	0.214	0.36 (0.14)	0.017
BMI	0.01 (0.01)	0.154	0.02 (0.02)	0.248	0.01 (0.02)	0.624
Unadjusted	0.13 (0.08)	0.116	0.30 (0.11)	0.006	0.52 (0.17)	0.003

Notes: Mandibular cortical bone was categorized using Klemetti´s Index as normal, moderate, or severe eroded cortex and the trabeculation classified into sparse or normal (mixed or dense); Education included all three levels namely low, medium, and high.

[a)] Mean height loss was estimated from 12- year follow-up period, [b)] Mean height loss was estimated from 13-year follow-up period.

For peer review only

For peer review only - http://bmjopen.bmj.com/site/about/guidelines.xhtml

The importance of the covariates for predicting height loss are shown in Table 3. Age is by far the most important parameter. Physical activity was predictive of height loss in some but not all periods, independently of bone structure. Baseline height, smoking and education showed inconsistent associations with height loss in different models, whereas BMI was not significant in any period.

DISCUSSION

Statement of principal findings

The present study was conducted among three systematically recruited birth cohorts from the Prospective Population Study of Women in Gothenburg, Sweden. We found that the longitudinal height loss was significantly predicted by mandibular bone parameters such as severe erosion of the cortex and sparse trabeculation. There was a clear increase in height loss over the follow-up time, in parallel with an increasing cortical erosion and sparseness of trabeculae. Severe cortical erosion was the better risk factor for height loss in the first two periods when the participants were younger, and the trabecular sparseness was a better predictor in the last period corresponding to the oldest ages.

Our findings can be applicable to many settings. Swedish and Norwegian women have the highest frequencies of fragility fractures in the world (26); i.e., they probably have sparser trabeculation and more severe cortical erosion than women in other countries, but if for instance Japanese women have severe eroded cortex (15), they may be of risk for height loss as well. Our main finding that severe cortical erosion and sparse trabeculation predict height loss builds on previous work in these cohorts showing mandibular bone structure to be a risk factor for future fractures (23,27,28).

For peer review only - http://bmjopen.bmj.com/site/about/guidelines.xhtml

In the first two examination periods, physical activity at the baseline of the study was protective against height loss, which is also in line with other studies (20, 29). On the other hand, smoking habits at the baseline had an association only in the last period where the participants were the oldest, in contrast to other studies that have shown strong associations.

Comparisons with other studies

Many previous studies have used height as a proxy for health to compare the welfare of populations, whereas few have investigated how not only height but also height loss could be associated with health in old age (9, 30, 31). Height is considered a marker of early life health, whereas height loss is a potential risk factor for later life health (9, 30, 31).

Huang et al. 2013 estimated pre-shrinkage height and studied the association between height loss and different measures of health of older people (9). They found that height loss for both men and women was negatively associated with better education, urban residence, and household economy. Moreover, height loss was positively correlated with poor health outcomes, especially strong for cognition decline (9). Fernihough & McGovern found that height loss among older English men and women across a lifetime decreases by 2 cm to 4 cm on average (30). These number are similar to our findings, where we found an average decrease of 4.3 cm after 37 years.

Jain & Ma 2020 found that mid-aged and older Indonesian women exhibit considerable height loss despite being short to begin with, losing an average of 1.06 cm over 17 years. Ethnicity, working in the agricultural sector and availability of local health infrastructure were correlated with loss of height (31). Extreme height loss, greater than 3 cm, was associated with 8-10 percent lower lung function and grip strength and with an increased mortality (31). They also found that loss of height is higher among older age groups and taller individuals (31), which is in accordance with our results. A recent investigation demonstrated, using

For peer review only - http://bmjopen.bmj.com/site/about/guidelines.xhtml

national register data linked to the same cohort as described here, that loss of height over 12 years predicted mortality from all-cause and cardiovascular disease (4). This is in line with the study of Jain & Ma (31). Such data is not widely available in prospective epidemiological studies.

Height loss is associated with poor health (1,4,7,8,9,29-31). Vertebral discs degenerate far earlier than other musculoskeletal tissues (1) and can lead to back pain and disability in the older adults (1) and in severe cases even vertebral fractures, which have been shown to have direct or indirect consequences such as decreased mobility, depression, hospitalization, and mortality (21).

Panoramic dental radiography indices, cortical thickness, cortical erosion, and sparse trabeculation, have been suggested as screening tools for identifying risk groups with osteoporosis (15,16,23). Cortical erosion, assessed at dental practices, has been shown to be useful in the identification of undetected low bone mineral density and spine fractures among postmenopausal women (16,17). However, Okabe et al. 2008 found no significant correlations between cortical erosion assessments and the occurrence of fractures in a group of 80-years old Japanese men and women (14). The difference in the results may be attributed to ethnicity-related anthropometric differences, like height, and the high frequencies of fragility fractures in Swedish and Norwegian women (26). Another difference is that Okabe et al. studied five years of fracture incidence whereas Jonasson et al investigated the same over ten years (14).

Mandibular cortical width is associated with general bone mineral density (15,19,32). A previous study in this cohort of Swedish women at ages 62 to 78 years investigated mandibular cortical erosion and thickness as predictors of future osteoporotic fractures and found that severe eroded cortices predicted fractures, but cortical width did not (27). Sparse

For peer review only - http://bmjopen.bmj.com/site/about/guidelines.xhtml

trabeculation was shown to be associated with a twofold higher risk for future fracture in a younger group and with a three- to fourfold higher risk for future fracture in an older group (28). As far as we know, relationships between height loss and oral indices have not been examined before.

All bone structures undergo an alteration in the bone remodeling mechanism in connection with menopause, leading to greater bone loss than bone formation (33). The imbalance results in trabecular thinning, increase in intra-trabecular spacing, loss of connectivity in the meshwork and intra-cortical erosion, thereby leading to diminished bone strength and increased risk of fracture, observed with increasing age (34,35). In long bones, a thin cortex and porosity, along with the body height have been shown to be associated with fracture risk (36). In that study, taller women had thinner cortical bone (in the appendicular skeleton) and therefore had a greater risk for fractures. This, in general, could also be applied to the pathophysiology behind cortical erosion in the vertebra (36).

A study has described the differences between mandibular versus long bones in terms of osteogenic potential and bone homeostasis (37). Despite the differences in embryological origin, menopause related cortical thinning should be an expected finding in the jawbones as well (37). An experimental study examined differences in the osteoclastic process at different sites of the body. Results showed no significant difference in terms of resorption rate between long bones and jawbones, although larger (yet fewer) osteoclasts were detected in the jawbones (38).

The vertebral bone mass is largely trabecular with only a thin shell of cortical bone. The trabecular bone is remodeled and lost more rapidly than the cortical bone, but later in life, the main bone loss occurs in the cortical compartment (36). With aging, progressive bone loss leads to less dense bone pattern. Therefore, the axial compressive load on the vertebra is

For peer review only - http://bmjopen.bmj.com/site/about/guidelines.xhtml

distributed on fewer and thinner trabeculae, resulting in an increasing risk for fractures (39,40).

Strengths and limitations

The strengths are the prospective design, the population-based sample, the long follow-up and that our findings can be applicable to many settings since many individuals visit their dentist at least every two years and radiographs are taken. A unique aspect of this study is its design, consisting of long-term panel data on bone structure and height. However, this study is not without limitations, such as the fact that height loss reflects various conditions that may make accurate height measures difficult, Moreover, participation rates became lower in the later years, and the drop out for different reasons, mortality included, probably differs between age groups, which might impact the mean height losses and the associations to covariates.

Implications of the findings

In our study population, the sparse trabeculation and the severe cortical erosion of the mandible could be seen to mimic the status of the vertebral trabeculae and intracortical erosion of the thin cortical shell of the vertebrae, which would, in turn, explain the potential height reduction. These two bone-related risk factors are important for the development of height loss as well as osteoporosis. A vertebral fracture is often asymptomatic, but it should not be ignored since it is a highly significant risk factor for future fractures, and if symptomatic, it is difficult to relieve the patients suffering.

Visual assessments of the mandibular trabecular and cortical bone as conducted in this study are not routine in dental practice today, but simplified screening procedures using artificial intelligence may change this in the future (41). Cortical erosion is easily evaluated using panoramic radiographs, whereas the trabeculation is best assessed on periapical radiographs. Bone turnover is probably the highest in the mandibular alveolar process (42),

For peer review only - http://bmjopen.bmj.com/site/about/guidelines.xhtml

and therefore, these results highlight the need for implementation studies in in which dental practitioners can become active participants in identifying individuals at risk of shrinkage, osteoporosis, and fractures, in collaboration with other clinicians.

CONCLUSION

The mandibular bone structure alterations such as severe cortical erosion and sparse trabeculation predicted height loss. They may therefore serve as proxy indicators when screening in the early phases of bone degenerative pathogenesis, signaling the ongoing bone remodeling and the need for further clinical attention to the older women at risk for height loss.

Contributors: NNG and LL initiated the study. VS and GJ did the acquisition of data. NNG, LL, VS, GJ and ER contributed to the analysis and interpretation of data. VS and NNG did the statistical analyses. NNG wrote the first draft of the manuscript. NNG, LL, GJ, ER and VS critically revised the manuscript and approved the final version. LL obtained the funding.

Competing interest: None

Funding: This work was supported by grants from the Swedish Research Council for Health, Wing Life and Welfare (Forte, EpiLife 200i6-1506), and the Swedish ALF-agreement (ALFGBG 720201).

Data sharing statement: No additional data available.

Ethics Approval: The Prospective Population Study of Women in Gothenburg has been approved by the Regional Ethics Review Board of the University of Gothenburg (*DNR 65-80, 179-92, T453-04*). The examinations comply with the Declaration of Helsinki and informed consent has been obtained from the subjects.

For peer review only - http://bmjopen.bmj.com/site/about/guidelines.xhtml

For peer review only

For peer review only - http://bmjopen.bmj.com/site/about/guidelines.xhtml

References

1. Hillier TA, Lui LY, Kado DM, LeBlanc ES, Vesco KK, Bauer DC, et al. Height loss in older women: risk of hip fracture and mortality independent of vertebral fractures. *J Bone Miner Res*. 2012;27:153-159. doi: 10.1002/jbmr.558
2. Gavriilidou NN, Pihlsgard M, Elmstahl S. Anthropometric reference data for elderly Swedes and its disease-related pattern. *Eur J Clin Nutr.* 2015;69:1066-1075. doi: 10.1038/ejcn.2015.73.
3. Pluskiewicz W, Adamczyk P, Drozdzowska B. The significance of height loss in postmenopausal women. The results from GO Study. *Int J Clin Pract.* 2021;75:e14009. doi: 10.1111/ijcp.14009.
4. Klingberg S, Mehlig K, Dangol R, Björkelund C, Heitmann BL, Lissner L. Loss of height predicts total and cardiovascular mortality: a cohort study of northern European women. *BMJ Open.* 2021;11:e049122. doi: 10.1136/bmjopen-2021-049122.
5. Perissinotto E, Pisent C, Sergi G, Grigoletto F, Group IW. Anthropometric measurements in the elderly: age and gender differences. *Br J Nutr.* 2002;87:177-186. doi: 10.1079/bjn2001487.
6. Gavriilidou NN, Pihlsgard M, Elmstahl S. High degree of BMI misclassification of malnutrition among Swedish elderly population: Age-adjusted height estimation using knee height and demispan. *Eur J Clin Nutr.* 2015;69:565-571. doi: 10.1038/ejcn.2014.183.
7. Urban JP, Roberts S. Degeneration of the intervertebral disc. *Arthritis Res Ther.* 2003;5:120-130. doi: 10.1186/ar629.
8. Wannamethee SG, Shaper AG, Lennon L, Whincup PH. Height loss in older men: associations with total mortality and incidence of cardiovascular disease. *Arch Intern Med.* 2006;166:2546-2552. doi: 10.1001/archinte.166.22.2546.

For peer review only - http://bmjopen.bmj.com/site/about/guidelines.xhtml

9. Huang W, Lei X, Ridder G, Strauss J, Zhao Y. Health, Height, Height Shrinkage, and SES at Older Ages: Evidence from China. *Am Econ J Appl Econ.* 2013;5:86-121. doi: 10.1257/app.5.2.86

10. Kanis JA. Assessment of fracture risk and its application to screening for postmenopausal osteoporosis: synopsis of a WHO report. WHO Study Group. *Osteoporos Int*. 1994;4:368-381. doi: 10.1007/BF01622200

11. Jonasson G, Bankvall G, Kiliaridis S. Estimation of skeletal bone mineral density by means of the trabecular pattern of the alveolar bone, its interdental thickness, and the bone mass of the mandible. *Oral Surg Oral Med Oral Pathol Oral Radiol Endod.* 2001;92:346-352. doi: 10.1067/moe.2001.116494.

12. Lindh C, Horner K, Jonasson G, Olsson P, Rohlin M, Jacobs R, et al. The use of visual assessment of dental radiographs for identifying women at risk of having osteoporosis: the OSTEODENT project. *Oral Surg Oral Med Oral Pathol Oral Radiol Endod.* 2008;106:285-293. doi: 10.1016/j.tripleo.2007.09.008.

13. Lindh C, Petersson A, Rohlin M. Assessment of the trabecular pattern before endosseous implant treatment: diagnostic outcome of periapical radiography in the mandible. *Oral Surg Oral Med Oral Pathol Oral Radiol Endod*. 1996;82:335-243. doi: 10.1016/s1079-2104(96)80363-5

14. Okabe S, Morimoto Y, Ansai T, Yoshioka I, Tanaka T, Taguchi A, et al. Assessment of the relationship between the mandibular cortex on panoramic radiographs and the risk of bone fracture and vascular disease in 80-year-olds. *Oral Surg Oral Med Oral Pathol Oral Radiol Endod.* 2008;106:433-424. doi: 10.1016/j.tripleo.2007.09.013.

15. Taguchi A. Panoramic radiographs for identifying individuals with undetected osteoporosis. *Japanese Dental Science Review.* 2009;45:109-120. doi.org/10.1016/j.jdsr.2009.05.001

For peer review only - http://bmjopen.bmj.com/site/about/guidelines.xhtml

16. Taguchi A, Ohtsuka M, Nakamoto T, Naito K, Tsuda M, Kudo Y, et al. Identification of post-menopausal women at risk of osteoporosis by trained general dental practitioners using panoramic radiographs. *Dentomaxillofac Radiol*. 2007;36:149-154. doi: 10.1259/dmfr/31116116.

17. Lissner L, Skoog I, Andersson K, Beckman N, Sundh V, Waern M, et al. Participation bias in longitudinal studies: experience from the Population Study of Women in Gothenburg, Sweden. *Scand J Prim Health Care.* 2003;21:242-247. doi: 10.1080/02813430310003309-1693.

18. Bengtsson C, Ahlqwist M, Andersson K, Bjorkelund C, Lissner L, Soderstrom M. The Prospective Population Study of Women in Gothenburg, Sweden, 1968-69 to 1992-93. A 24-year follow-up study with special reference to participation, representativeness, and mortality. *Scand J Prim Health Care.* 1997;15:214-219. doi: 10.3109/02813439709035031.

19. Klemetti E, Kolmakov S, Kroger H. Pantomography in assessment of the osteoporosis risk group. *Scand J Dent Res*. 1994;102:68-72. doi: 10.1111/j.1600-0722.1994.tb01156.x.

20. Sagiv M, Vogelaere PP, Soudry M, Ehrsam R. Role of physical activity training in attenuation of height loss through aging. *Gerontology*. 2000;46:266-270. doi: 10.1159/000022170.

21. Xu W, Perera S, Medich D, Fiorito G, Wagner J, Berger LK, et al. Height loss, vertebral fractures, and the misclassification of osteoporosis. *Bone.* 2011;48:307-311. doi: 10.1016/j.bone.2010.09.027.

22. Thornton MJ, Sedlak CA, Doheny MO. Height change and bone mineral density: revisited. *Orthop Nurs.* 2004;23:315-320. doi: 10.1097/00006416-200409000-00007.

For peer review only - http://bmjopen.bmj.com/site/about/guidelines.xhtml

23. Jonasson G, Sundh V, Ahlqwist M, Hakeberg M, Bjorkelund C, Lissner L. A prospective study of mandibular trabecular bone to predict fracture incidence in women: a low-cost screening tool in the dental clinic. *Bone.* 2011;49:873-879. doi: 10.1016/j.bone.2011.06.036.

24. Grimby G, Börjesson M, Jonsdottir IH, Schnohr P, Thelle DS, Saltin B. The "Saltin-Grimby Physical Activity Level Scale" and its application to health research. *Scand J Med Sci Sports.* 2015;25 Suppl 4:119-25. doi: 10.1111/sms.12611

25. Kaufman JS, Cooper RS. Seeking Causal Explanations in Social Epidemiology. *Am J Epidemiol.* 1999;150:113-120. doi: 10.1093/oxfordjournals.aje.a009969.

26. SBU. Osteoporos – prevention, diagnostik och behandling. En systematisk litteraturöversikt. Stockholm.: Statens beredning för medicinsk utvärdering (SBU). 2003.

27. Jonasson GB, Sundh V, Hakeberg M, Ahlqwist M, Lissner L, Hange D. Evaluation of clinical and radiographic indices as predictors of osteoporotic fractures: a 10-year longitudinal study. *Oral Surg Oral Med Oral Pathol Oral Radiol.* 2018;125:487-494. doi: 10.1016/j.oooo.2017.11.009.

28. Sundh V, Hange D, Ahlqwist M, Hakeberg M, Lissner L, Jonasson G. FRAX and mandibular sparse trabeculation as fracture predictors: a longitudinal study from 1980 to 2002. *Eur J Oral Sci.* 2017;125:135-140. doi: 10.1111/eos.12341.

29. Mai X, Marshall B, Hovey KM, Sperrazza J, Wactawski-Wende J. Risk factors for 5-year prospective height loss among postmenopausal women. *Menopause.* 2018;25:883-889. doi: 10.1097/GME.0000000000001108.

30. Fernihough A, McGovern ME. Physical Stature Decline and the Health Status of the Elderly Population in England. *Econ Hum Biol.* 2015;16:30-44. doi:10.1016/j.ehb.2013.12.010.

For peer review only - http://bmjopen.bmj.com/site/about/guidelines.xhtml

31. Jain U, Ma M. Height shrinkage, health and mortality among older adults: Evidence from Indonesia. *Econ Hum Biol*. 2020;37:100863. doi: 10.1016/j.ehb.2020.100863.

32. Devlin H, Horner K. Mandibular radiomorphometric indices in the diagnosis of reduced skeletal bone mineral density. *Osteoporos Int.* 2002;13:373-378. doi: 10.1007/s001980200042.

33. Lerner UH. Bone remodeling in post-menopausal osteoporosis. *J Dent Res.* 2006;85:584-595. doi: 10.1177/154405910608500703.

34. Bala Y, Zebaze R, Seeman E. Role of cortical bone in bone fragility. *Curr Opin Rheumatol.* 2015;27(4):406-13. doi: 10.1097/BOR.0000000000000183.

35. Bjornerem A, Wang X, Bui M, Ghasem-Zadeh A, Hopper JL, Zebaze R, et al. Menopause-Related Appendicular Bone Loss is Mainly Cortical and Results in Increased Cortical Porosity. *J Bone Miner Res.* 2018;33:598-605. doi: 10.1002/jbmr.3333.

36. Bjornerem A, Bui QM, Ghasem-Zadeh A, Hopper JL, Zebaze R, Seeman E. Fracture risk and height: an association partly accounted for by cortical porosity of relatively thinner cortices. *J Bone Miner Res.* 2013;28:2017-2026. doi: 10.1002/jbmr.1934.

37. Aghaloo TL, Chaichanasakul T, Bezouglaia O, Kang B, Franco R, Dry SM, et al. Osteogenic potential of mandibular vs. long-bone marrow stromal cells. *J Dent Res* 2010;89:1293-1298. doi: 10.1177/0022034510378427..

38. de Souza Faloni AP, Schoenmaker T, Azari A, Katchburian E, Cerri PS, de Vries TJ, et al. Jaw and long bone marrows have a different osteoclastogenic potential. *Calcif Tissue Int.* 2011;88:63-74. doi: 10.1007/s00223-010-9418-4.

39. Adams MA, Hutton WC. The Mechanical Function of the Lumbar Apophyseal Joints. *Spine.* 1983;8. doi: 10.1097/00007632-198304000-00017.

For peer review only - http://bmjopen.bmj.com/site/about/guidelines.xhtml

40. Giambini H, Salman Roghani R, Thoreson AR, Melton LJ, 3rd, An K-N, Gay RE. Lumbar trabecular bone mineral density distribution in patients with and without vertebral fractures: a case-control study. *Eur Spine J.* 2014;23:1346-1353. doi: 10.1007/s00586-014-3205-2.

41. Jonasson G, Skoglund I, Rythén M. The rise and fall of the alveolar process: Dependency of teeth and metabolic aspects. *Arch Oral Biol*. 2018;96:195-200. https://doi.org/10.1016/j.archoralbio.2018.09.016

42. Jonasson G, Billhult A. Mandibular bone structure, bone mineral density, and clinical variables as fracture predictors: a 15-year follow-up of female patients in a dental clinic. *Oral Surg Oral Med Oral Pathol Oral Radiol.* 2013;116:362-368. doi: 10.1016/j.oooo.2013.06.009.

For peer review only

Figure legend

Figure 1: Flow chart.

For peer review only

For peer review only - http://bmjopen.bmj.com/site/about/guidelines.xhtml

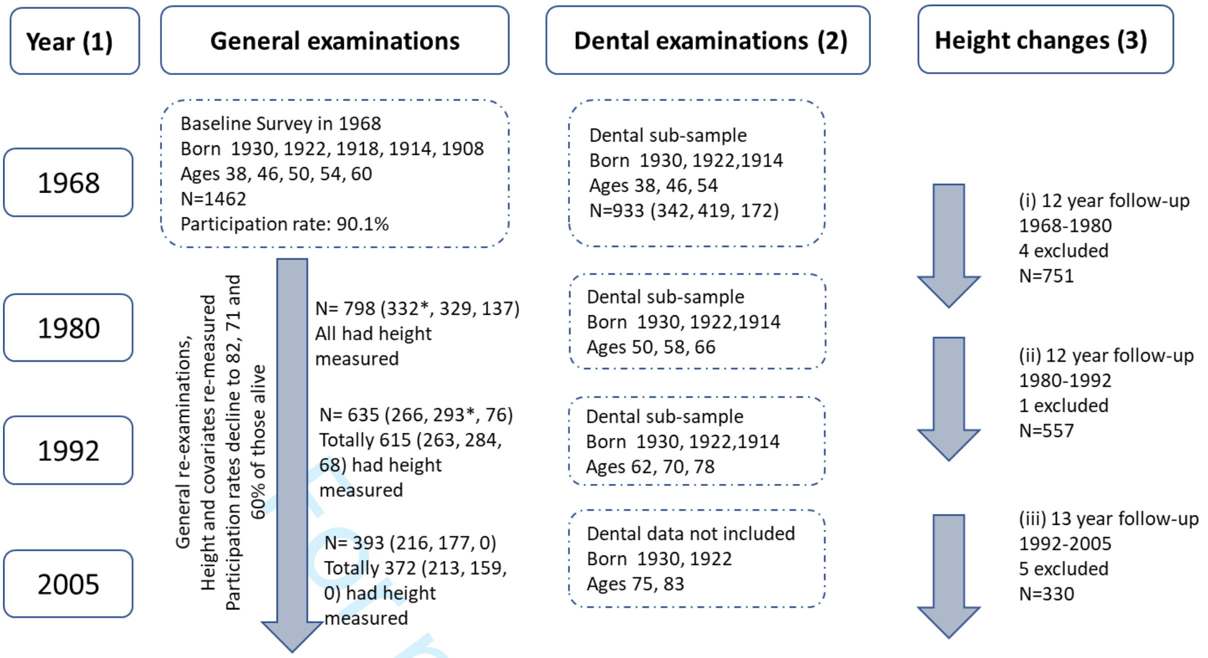

(1) Each examination period starting in September and ending in June of following year, i.e., 1968-69, 1980-81, 1992-93, 2005-06
(2) Dental examination conducted shortly after general examination in three of original age groups. Mandibular bone structure (cortical and trabecular bone) is the main independent variable
(3) Heights were measured at general examinations, and the change in height was estimated at 12- or 13-year intervals, after excluding any increases of over 2 cm

STROBE 2007 (v4) Statement—Checklist of items that should be included in reports of *cohort studies*

Section/Topic	Item #	Recommendation	Reported on page #
**Title and abstract**	1	(*a*) Indicate the study's design with a commonly used term in the title or the abstract	1 and 2
		(*b*) Provide in the abstract an informative and balanced summary of what was done and what was found	2
**Introduction**			
Background/rationale	2	Explain the scientific background and rationale for the investigation being reported	5
Objectives	3	State specific objectives, including any prespecified hypotheses	6
**Methods**			
Study design	4	Present key elements of study design early in the paper	2-5
Setting	5	Describe the setting, locations, and relevant dates, including periods of recruitment, exposure, follow-up, and data collection	2, 5
Participants	6	(*a*) Give the eligibility criteria, and the sources and methods of selection of participants. Describe methods of follow-up	5
		(*b*) For matched studies, give matching criteria and number of exposed and unexposed	-
Variables	7	Clearly define all outcomes, exposures, predictors, potential confounders, and effect modifiers. Give diagnostic criteria, if applicable	7, 8
Data sources/ measurement	8*	For each variable of interest, give sources of data and details of methods of assessment (measurement). Describe comparability of assessment methods if there is more than one group	*7, 8*
Bias	9	Describe any efforts to address potential sources of bias	11, 12
Study size	10	Explain how the study size was arrived at	7
Quantitative variables	11	Explain how quantitative variables were handled in the analyses. If applicable, describe which groupings were chosen and why	8
Statistical methods	12	(*a*) Describe all statistical methods, including those used to control for confounding	8
		(*b*) Describe any methods used to examine subgroups and interactions	8, 10
		(*c*) Explain how missing data were addressed	6
		(*d*) If applicable, explain how loss to follow-up was addressed	-
		(*e*) Describe any sensitivity analyses	-
**Results**			

For peer review only

Participants	13*	(a) Report numbers of individuals at each stage of study—eg numbers potentially eligible, examined for eligibility, confirmed eligible, included in the study, completing follow-up, and analysed	6, 7 +fig 1
		(b) Give reasons for non-participation at each stage	6, 7
		(c) Consider use of a flow diagram	Fig 1
Descriptive data	14*	(a) Give characteristics of study participants (eg demographic, clinical, social) and information on exposures and potential confounders	6, 7
		(b) Indicate number of participants with missing data for each variable of interest	Table 1
		(c) Summarise follow-up time (eg, average and total amount)	Fig 1 + tables
Outcome data	15*	Report numbers of outcome events or summary measures over time	5-8
Main results	16	(*a*) Give unadjusted estimates and, if applicable, confounder-adjusted estimates and their precision (eg, 95% confidence interval). Make clear which confounders were adjusted for and why they were included	Tables
		(*b*) Report category boundaries when continuous variables were categorized	Tables
		(*c*) If relevant, consider translating estimates of relative risk into absolute risk for a meaningful time period	-
Other analyses	17	Report other analyses done—eg analyses of subgroups and interactions, and sensitivity analyses	-
**Discussion**			
Key results	18	Summarise key results with reference to study objectives	10
**Limitations**			
Interpretation	20	Give a cautious overall interpretation of results considering objectives, limitations, multiplicity of analyses, results from similar studies, and other relevant evidence	14
Generalisability	21	Discuss the generalisability (external validity) of the study results	2, 11
**Other information**			
Funding	22	Give the source of funding and the role of the funders for the present study and, if applicable, for the original study on which the present article is based	15

*Give information separately for cases and controls in case-control studies and, if applicable, for exposed and unexposed groups in cohort and cross-sectional studies.

Note: An Explanation and Elaboration article discusses each checklist item and gives methodological background and published examples of transparent reporting. The STROBE checklist is best used in conjunction with this article (freely available on the Web sites of PLoS Medicine at http://www.plosmedicine.org/, Annals of Internal Medicine at http://www.annals.org/, and Epidemiology at http://www.epidem.com/). Information on the STROBE Initiative is available at www.strobe-statement.org.

For peer review only

BMJ Open

Does mandibular bone structure predict subsequent height loss? A longitudinal cohort study of women in Gothenburg, Sweden

Journal:	*BMJ Open*
Manuscript ID	bmjopen-2022-066844.R2
Article Type:	Original research
Date Submitted by the Author:	21-Feb-2023

Complete List of Authors:	Gavriilidou, Nivetha N; Karolinska Institute, Department of Dental Medicine<br>Jonasson, Grethe; University of Gothenburg, Department of Behavioral and Community Dentistry<br>Sundh, Valter; University of Gothenburg, School of Public Health and Community Medicine, Institute of Medicine, Sahlgrenska Academy,<br>Rothenberg, Elisabet ; Kristianstad University, Department of Nursing and Integrated Health Sciences<br>Lissner, Lauren; University of Gothenburg Sahlgrenska Academy, School of Public Health and Community Medicine, Institute of Medicine, Sahlgrenska Academy,

**Primary Subject Heading**:	Dentistry and oral medicine
Secondary Subject Heading:	Dentistry and oral medicine
Keywords:	ORAL MEDICINE, Bone diseases < ORTHOPAEDIC & TRAUMA SURGERY, PUBLIC HEALTH

SCHOLARONE™
Manuscripts

I, the Submitting Author has the right to grant and does grant on behalf of all authors of the Work (as defined in the below author licence), an exclusive licence and/or a non-exclusive licence for contributions from authors who are: i) UK Crown employees; ii) where BMJ has agreed a CC-BY licence shall apply, and/or iii) in accordance with the terms applicable for US Federal Government officers or employees acting as part of their official duties; on a worldwide, perpetual, irrevocable, royalty-free basis to BMJ Publishing Group Ltd ("BMJ") its licensees and where the relevant Journal is co-owned by BMJ to the co-owners of the Journal, to publish the Work in this journal and any other BMJ products and to exploit all rights, as set out in our licence.

The Submitting Author accepts and understands that any supply made under these terms is made by BMJ to the Submitting Author unless you are acting as an employee on behalf of your employer or a postgraduate student of an affiliated institution which is paying any applicable article publishing charge ("APC") for Open Access articles. Where the Submitting Author wishes to make the Work available on an Open Access basis (and intends to pay the relevant APC), the terms of reuse of such Open Access shall be governed by a Creative Commons licence – details of these licences and which Creative Commons licence will apply to this Work are set out in our licence referred to above.

Other than as permitted in any relevant BMJ Author's Self Archiving Policies, I confirm this Work has not been accepted for publication elsewhere, is not being considered for publication elsewhere and does not duplicate material already published. I confirm all authors consent to publication of this Work and authorise the granting of this licence.

For peer review only

Does mandibular bone structure predict subsequent height loss? A longitudinal cohort study of women in Gothenburg, Sweden

Nivetha N Gavriilidou DDS MPH PhD, [a] Grethe Jonasson DDS PhD, [b] Valter Sundh BSc, [c] Elisabet Rothenberg RD PhD, [d] Lauren Lissner MPH PhD. [e]

[a] Karolinska Institute, Department of Dental Medicine, Institute of Odontology, Karolinska Institute, Sweden. Email: nivetha.gavriilidou@ki.se

[b] Department of Behavioral and Community Dentistry, Institute of Odontology at the Sahlgrenska Academy, University of Gothenburg, Sweden. Email: grethe.jonasson@gu.se

[c.] School of Public Health and Community Medicine, Institute of Medicine, Sahlgrenska Academy, University of Gothenburg, Sweden. Email: valter.sundh@gu.se

[d] Department of Nursing and Integrated Health Sciences, Faculty of Health Sciences, Kristianstad University, Sweden. Email: elisabet.rothenberg@hkr.se

[e] School of Public Health and Community Medicine, Institute of Medicine, Sahlgrenska Academy, University of Gothenburg, Sweden. Email: lauren.lissner@gu.se

Corresponding author: Grethe Jonasson, Department of Behavioral and Community Dentistry, Institute of Odontology at the Sahlgrenska Academy, University of Gothenburg, Sweden. Email: grethe.jonasson@gu.se; tel +46 709285671

Short title: Mandibular bone structure and stature shrinkage.

For peer review only - http://bmjopen.bmj.com/site/about/guidelines.xhtml

ABSTRACT

Background Several risk factors for loss of height with increasing age have been identified.

Objective To investigate if mandibular bone structure predicts future height loss in middle-aged and elderly Swedish women.

Design Prospective cohort study with longitudinally measured heights, radiographical assessments of the cortical bone using Klemetti´s Index (normal, moderate, or severely eroded cortex), and classification of the trabecular bone using an index proposed by Lindh et al. (sparse, mixed, or dense trabeculation). No intervention was performed.

Setting Gothenburg, Sweden.

Participants A population-based sample of 937 Swedish women born in 1914, 1922 and 1930 was recruited. At the baseline examination, the ages were 38, 46, and 54. All had undergone a dental examination with panoramic radiographs of the mandible, and a general examination including height measurements on at least 2 occasions.

Main outcome measure Height loss was calculated over three 12-13-year periods (1968-80, 1980-92, 1992- 2005).

Main results. Mean annual height loss measures were 0.075cm/year, 0.08 cm/year, and 0.18 cm/year over the 3 observation intervals, corresponding to absolute decreases of 0.9 cm, 1.0 cm, and 2.4 cm. Cortical erosion in 1968 and 1980 significantly predicted height loss 12 years later. Sparse trabeculation in 1968, 1980 and 1992 also predicted significant shrinkage over 12 or 13 years. Multivariable regression analyses adjusting for baseline covariates such as height, birth year, physical activity, smoking, body mass index, and education yielded consistent findings.

For peer review only

Conclusion Mandibular bone structure characteristics such as severe cortical erosion and sparse trabeculation may serve as early risk factors for height loss. Since most individuals visit their dentist at least every two years and radiographs are taken, a collaboration between dentists and physicians may open opportunities for predicting future risk of height loss.

Keywords Ageing, body height, cortical bone, dental radiography, mandible, stature decline, trabecular bone, women's health.

For peer review only

For peer review only - http://bmjopen.bmj.com/site/about/guidelines.xhtml

Strengths and limitations of this study

- The strengths are the prospective design, the population-based sample, the long follow-up, repeated measurements of height for the same individuals, and that our findings can be applicable to many settings.
- The main limitation is that loss of height could reflect different conditions which may make accurate height estimates difficult.
- The participation rates became lower in the later years, and the drop out for different reasons, mortality included, probably differs between age groups, which might impact the mean height losses.

For peer review only

For peer review only - http://bmjopen.bmj.com/site/about/guidelines.xhtml

INTRODUCTION

Height loss in older women is incrementally more pronounced at ages over 75 years. It has been found to be associated with increased morbidity and mortality (1-5). Several causes have been identified that lead to loss of height among older adults. Height loss could indicate age-associated progressive skeletal deformation such as kyphoscoliosis, flattening of plantar arch and altered posture (6,7), and/or degenerative processes including osteoporosis, vertebral compression, attrition of intervertebral discs, and vertebral fractures (8). These alterations make accurate height estimation a challenge. Comorbidities of major height loss in older adults include functional impairments such as dependence in daily life activities, cognitive impairment, and increased overall mortality risk (1,5). Height loss over 5cm has been shown to predict non-spinal fractures such as hip fractures and mortality independent of vertebral fractures (1,6,7,9,10). Height loss in women exceeding 4 cm has been found to be related to fracture probability of over 50% (3). In men, health consequences of height loss over 3 cm include increased risk of all-cause mortality and coronary heart disease (9).

Osteoporosis is a widely acknowledged risk factor for future fractures in the elderly. According to the World Health Organization, it is defined as bone density lower than 2.5 standard deviations under that of young women using the bone mineral density of the hip with dual X-ray absorptiometry (DXA) (11). Simple, practical, and relevant techniques are needed for early identification of the onset or extent of skeletal degeneration. Therefore, researchers have used oral variables such as optical density (12), sparse trabeculation (12-14), cortical width (15), and cortical erosion (15-17) for the prediction of osteoporosis and fracture.

Intraoral radiographs are usually an integral part of the routine oral examination. Assessment of mandibular structure, the cortical and trabecular bone pattern may be studied through visual inspection, hence providing a simple screening method for the evaluation of the bone condition. The hypothesis of the present study is that the mandibular bone contains

For peer review only - http://bmjopen.bmj.com/site/about/guidelines.xhtml

For peer review only

sufficient information about the general bone condition to predict future height loss. The objective is to investigate two proxies for general bone mineral density, namely mandibular cortical erosion, and trabecular coarseness, as predictors of height loss over subsequent periods of 12-13 years.

METHODS

Study population

The Prospective Population Study of Women in Gothenburg, Sweden, is an ongoing longitudinal study initiated in 1968 in a cohort of women aged 38, 46, 50, 54, or 60 years. At that time, a representative sample of women in Gothenburg was identified using the Revenue Office register with respect to the dates of birth. From the full baseline cohort, those born in 1914, 1922, and 1930 were included in the present analytic sample (flowchart, Figure 1). The original survey included general health and dental examinations, which were repeated over the years. Each wave of survey was started in the fall and completed in the spring of the following year, i.e., 1968-69, 1980-81, 1992-93, and 2004-05, and is hereafter referred to by its starting year. The participation rate in the initial examination was over 90% (18,19). A non-participation analysis of the 24-year follow-up in 1992 showed that the women who declined to participate in the first survey did not differ significantly from participants except for the long-term survival, which was lower in the initial refusers. The sample remained representative of the general population in terms of parameters such as blood pressure, weight, height, BMI, waist and hip circumference, serum cholesterol, triglyceride, and urate (19).

Inclusion criteria

In total, 937 women born in 1914, 1922 or 1930, were included in the present analysis. The inclusion criteria were availability of at least one panoramic radiograph in 1968, 1980, or

For peer review only

1992 from which it was possible to assess the mandibular cortex erosion and sparsity of trabeculae, plus availability of a baseline and at least one subsequent height measure. In the first period (1968-1989), four individuals were excluded due to an *increase* in height of at least 2 cm. Number excluded 1980-1992 was one, and it was five in the period 1992-2005. Results from previous observational studies using the same cohorts have shown that the number of participants should be sufficient for studying common health endpoints.

Patient and Public Involvement

No patients involved.

Main outcome

The main outcome variable was height loss during the 12-13 years following the dental examination. At all examinations, height and weight were assessed by standard anthropometric measurement techniques. A stadiometer was used to measure the participants' body heights (without shoes). Measurement of height was generally performed early in the day. Height loss was calculated as baseline height minus height at follow-up. Thus, a positive value indicated height loss.

Height change was assessed from follow-up examinations 12-13 years later, until 2005. The exclusion criterion was an observed *increase* in height of at least 2 cm during any of these follow-up periods, attributable to probable measurement errors.

There were three 12–13-year follow-up periods namely 1968-1980, 1980-1992, and 1992-2005, where the first period comprised middle-aged women initially aged 38, 46, 54 years, the second period with a subset of these women from ages 62, 70 and 78 years, and the third and the latest period starting at ages 75 and 83 years (no follow-up from 1914 birth cohort) (Figure 1).

Bone structure and other covariates

For peer review only - http://bmjopen.bmj.com/site/about/guidelines.xhtml

Radiographic assessment of mandibular bone structures was included as part of the dental examination. In the present analysis, the main independent variables were two components of mandibular bone status. On visual inspection of the panoramic radiograph of a mandible, the cortical bone was categorised using Klemetti´s Index as mild/none (normal cortex), moderate, or severely eroded cortex (20). A normal cortex means an even endosteal margin; a moderate eroded cortex has semilunar defects in the endosteal margin, whereas a severely eroded cortex has heavy endosteal cortical porosities. The second component, the trabeculation, was classified using an index proposed by Lindh et al. 1996 as sparse, mixed, or dense (13,14). Sparse trabeculation means large intertrabecular spaces in the premolar area. Dense trabeculation has small intertrabecular spaces. Mixed dense and sparse trabeculation is mostly dense at the crest and sparse apically.

Baseline variables such as weight, height, body mass index (BMI), smoking, physical activity, and education, were considered potential covariates that could be associated with mandibular erosion and trabecular pattern, as well as height loss (21-24). The survey questionnaire regarding smoking habit had 6 possible responses: Never smoked (0), Previous smoker but quit in the last 15 years (1), Previous smoker but quit since last year (2), Quit smoking since last year (3), Current smoker without deep inhalation of the smoke (4), and Current smoker with deep inhalation of the smoke (5). This variable was dichotomised to "*No smoking*" (0) including response options 0, 1, 2, and "*Smoking*" (1) including options 3, 4, or 5. The variable physical activity was categorised as (i) almost no physical activity or none (sedentary), (ii) at least 4 hours/ a week of physical activity, (iii) regular physical activity and (iiii) regular intense training and competitions (25). This variable was dichotomised as: "*physically inactive*" (0) including the first response and "*physically active*" (1) including the latter three choices. Education was considered a proxy for socioeconomic status as proposed

For peer review only - http://bmjopen.bmj.com/site/about/guidelines.xhtml

by Kaufmann (26) and categorised as low: pre-high school (up to 9 years); medium: high school with ≤2 years at university; and high: 3 or more years at university.

Statistical analyses

The mandibular bone variables are ordered categorical. Mann-Whitney Two-Sample Test was used for differences between two groups. In the analyses, the trabeculation variable was dichotomised into sparse and normal (mixed and dense). Cortical erosion has three categories. In table 2 normal cortex was reference category and tested against the two other categories. Height, height loss and BMI were continuous variables, whereas smoking and physical activity were dichotomised as described above; education was also a categorical variable with three categories. In order to test the importance of lifestyle factors, linear multiple regression analyses were performed, where height loss was the dependent variable. The following variables were independent covariates: the two mandibular bone variables, smoking, physical activity, height, body mass index (BMI), education, and age (birth year). BMI was considered as a measure of possible underweight/normal weight/overweight and therefore included. In the linear regression models, dichotomous variables were coded 0-1, and three categories of cortical erosion were coded 1-2-3. This is the most common way to analyse this variable (15, 16, 20). This three-level scale will produce an effect measure that is an averaged effect of the difference (in the outcome variable) between category 1 and 2 and between category 2 and 3, thereby testing the null hypothesis (mean_cat1 = mean_cat2 = mean_cat3 against the alternative hypothesis that mean_cat1 < mean_cat2 and/or mean_cat2 < mean_cat3 (positive trend) or mean_cat1 > mean_cat2 and/or mean_cat2 > mean_cat3 (negative trend), thereafter, test of trend over three levels was used. The significance level was $p<0.05$). Statistical analyses were performed with SAS version 9.2 software (SAS Institute Inc., Cary, NC).

For peer review only - http://bmjopen.bmj.com/site/about/guidelines.xhtml

RESULTS

Selected characteristics of the sample are shown in Table 1. Mean age at the four height measurements (year 1968, 1980, 1992, and 2005) were 44.9, 56.9, 68.0, and 78.8 years respectively, corresponding to three periods of height change (1968-80, 1980-92, and 1992-2005). There was a rising trend in the proportion of women with severe cortical erosion from 3.2% among the participants in the period 1968-1980, to 11.1% in the period 1980-1992 and up to 49.8% in the period 1992-2005. Similarly, prevalence of sparse trabeculation increased from 21.8% among the participants 1968-1980, to 37.2% in 1980-1992 and up to 48.3% in the period 1992-2005. At the same time, the mean height loss over the three survey periods was 0.9 cm, 1.0 cm, and 2.4 cm, with corresponding annual decreases of 0.075, 0.08, and 0.18 cm/year (Table 1).

Table 1: Descriptive characteristics of the population sub-samples born in 1914, 1922 and 1930 at examination year 1968 followed up across periods 1968-80, 1980-92, 1992-2005.

	1968	1980	1992	2005
Age of women born 1930 years (N)	38 (342)	50 (289)	62 (251)	75 (190)
Born 1922	46 (419)	58 (327)	70 (239)	83 (140)
Born 1914	54 (172)	66 (135)	78 (67)	91 (0) [a]
	Total (933)	Total (751)	Total (557)	Total (330)
**Parameters at the end of the period**	**1968**	**1968-80**	**1980-92**	**1992-2005**
Mean age (SD)	44.9 (5.8)	56.9 (5.8)	68.0 (5.4)	78.8 (3.8)
Mean height in cm (SD)	163.9 (6.0)	163.0 (6.1)	162.1 (6.2)	160.4 (6.1)
Almost no physical activity N (%)	157 (16.8)	221 (29.4)	117 (21.0)	25 (9.3)
Active Smokers N (%)	308 (41.0)	257 (34.3)	115 (20.6)	30 (9.2)
Education level [b]				
Low	630 (67.7)	506 (67.4)	364 (65.4)	209 (63.3)
Medium	251 (27.0)	205 (27.3)	159 (28.5)	93 (28.2)
High	49 (5.3)	39 (5.2)	33 (5.9)	28 (8.5)
Mean body mass index (kg/m^2)	23.7 (3.7)	24.7 (3.9)	26.4 (4.2)	26.2 (4.5)
Weight (kg)	63.6 (10.8)	66.0 (11.2)	69.0 (11.9)	68.8 (11.8)
**Period 1968- 80**				
Mean height Loss (cm (SD))	0.90 (0.89)			
Mean annual height loss (cm/year)	0.075			
**Period 1980- 92**				
Mean height Loss (cm (SD))		1.0 (1.21)		
Mean annual height loss (cm/year)		0.08		
**Period 1992- 2005**				
Mean height Loss (cm (SD))			2.4 (1.54)	

For peer review only

Mean annual height loss (cm/year)				0.18
**Mandibular parameters at the start of the period**				
Cortical Erosion *N (%)*				
Mild/None	583 (65.2)	451 (62.9)	175 (33.6)	5 (8.2)
Moderate	283 (31.7)	243 (33.9)	288 (55.3)	128 (42,0)
Severely eroded	28 (3.1)	23 (3.2)	58 (11.1)	152(49.8)
Trabeculation *N (%)*				
Normal	650 (79.4)	516 (78.2)	307 (62.8)	154 (51.7)
Sparse	169 (20.6)	144 (21.8)	182 (37.2)	144 (48.3)

Notes: Cortical bone was classified using Klemetti´s Index as normal, moderate, or severely eroded cortex and the trabeculation classified into sparse or normal (mixed, or dense). Number excluded based on shrinkage values over period 1968-1980: 4, 1980-1992: 1, 1968-1992: 4, 1992-2005: 5.

[a]): There were none in this age group in the follow up period 1992-2005.

Not all participants with height measurements had an assessment of cortical erosion and even fewer had trabecular assessment due to edentulism.

[b]): Education level categories: Low: pre-high school level (up to 9 years), Medium: high school level with ≤2 years at university, High: 3 or more years at university.

Table 2 describes prospective analyses of how mandibular bone structure at the three age periods predicted height loss, without covariate adjustment.

Table 2: Height loss in relation to three categories of cortical erosion and two categories of trabecular spacing.

P-values from Mann-Whitney test (MW) and Test of trend over three levels (Lin)

	**1968-1980 N=751**			**1980-1992 N=557**			**1992-2015 N=330**		
**Cortical Erosion**	Mean(SD)	Rate/y	P	Mean SD	Rate/y	P	Mean SD	Rate/y	p
1. Mild/none (ref)	0.84(0.84)	0.07	-	0.61±0.93	0.05	-	2.48±1.42	0.19	-
2. Moderate (vs 1)	0.99(0.92)	0.08	0.046 (MW)	1.04±1.17	0.09	<0.001(MW)	2.21±1.22	0.17	0.34(MW)
3. Severely (vs 1)	1.46(0.75)	0.12	<0.001(MW)	1.62±1.15	0.13	<0.001(MW)	2.62±1.86	0.20	0.81(MW)
Trend 1-2-3			<0.001(Lin)			<0.001(Lin)			0.195(Lin)
**Trabecular Pattern**									
Normal	0.88±0.86	0.07		0.81±1.04	0.07		2.16±1.26	0.16	
Sparse	1.01±0.99	0.08	0.011 (MW)	1.11±1.15	0.09	0.04 (MW)	2.72±1.82	0.20	0.002(MW)

Notes: Mandibular cortical bone was classified using Klemetti´s Index as normal, moderate, or severely eroded cortex, and the trabeculation classified into sparse or normal (mixed or dense).

Height loss was highest in the groups with severe cortical erosion in the two first periods, whereas the group with sparse trabeculation had the highest bone loss during all three

For peer review only - http://bmjopen.bmj.com/site/about/guidelines.xhtml

observation periods. On average, the difference in shrinkage between the two groups with milder versus severe erosion was 0.62 cm in the early period, 0.99 cm in the middle period, and only 0.14 cm in the last period (Table 2). The difference in height loss between the group with normal/mixed and that with sparse trabeculation was 0.13 cm in the early period, 0.30 cm in the middle period, and 0.56 cm in the last period. In all the three periods, the sparse trabeculation group presented more height loss than that with normal or mixed trabeculation (Table 2). Multivariable linear regression analyses in Table 3 confirmed the significant associations between the height loss and cortical erosion during the periods 1968- 80 and 1980-92, but not 1992-2005, after adjusting for covariates as height, age (birth year), physical activity, smoking, education, and BMI. The trabeculation showed a significant correlation with height loss in all three periods (Table 3).

Table 3: Linear multivariable regression models predicting height loss over time intervals 1968-1980, 1980-1992 and 1992-2005. The prediction is based on cortical erosion(1,2,3) and trabeculation(0,1) at baseline (1968), adjusting for covariates height, year of birth, physical activity, smoking, education, and body mass index (BMI).

	**Height loss (1968-80) N= 751**		**Height loss (1980-92) N= 557**		**Height loss (1992-2005) N=330**	
	Coefficient (Std Error)	P value	Coefficient Std Error	P value	Coefficient Std Error	P value
**Cortical Erosion**	**0.14 (0.06)**	**0.013**	**0.30 (0,09)**	**<0.001**	**0.15 (0.15)**	**0.338**
Height at baseline	0.01 (0.01)	0.217	0.02 (0.01)	0.088	0.02 (0.02)	0.156
Birth year	-0.05 (0.01)	<0.001	-0.06 (0.01)	<0.001	-0.02 (0.02)	0.321
Physical activity	-0.10 (0.07)	0.437	-0.25 (0.12)	0.036	0.10 (0.19)	0.481
Smoking	-0.04 (0.06)	0.517	0.06 (0.11)	0.579	0.34 (0.19)	0.013
Education	-0.01 (0.05)	0.938	0.09 (0.09)	0.299	0.39 (0.15)	0.009
BMI	0.01 (0.01)	0.194	-0.01 (0.02)	0.828	0.012 (0.03)	0.490
Unadjusted	0.21 (0.06)	<0.001	0.45 (0.08)	<0.001	0.18 (0.14)	0.195
**Trabeculation**	**0.17 (0.08)**	**0.030**	**0.24 (0.12)**	**0.021**	**0.40 (0.19)**	**0.032**
Height at baseline	0.01 (0.01)	0.048	0.02 (0.01)	0.015	0.02 (0.02)	0.359
Birth year	-0.06 (0.01)	<0.001	-0.06 (0.01)	<0.001	-0.01 (0.02)	0.554
Physical activity	-0.24 (0.07)	<0.001	-0.26 (0.12)	0.026	-0.01 (0.26)	0.977
Smoking	-0.04 (0.07)	0.576	-0.07 (0.10)	0.509	0.33 (0.19)	0.076
Education	-0.03 (0.06)	0.553	0.11 (0.08)	0.214	0.36 (0.14)	0.017
BMI	0.01 (0.01)	0.154	0.02 (0.02)	0.248	0.01 (0.02)	0.624
Unadjusted	0.13 (0.08)	0.116	0.30 (0.11)	0.006	0.52 (0.17)	0.003

For peer review only - http://bmjopen.bmj.com/site/about/guidelines.xhtml

For peer review only

Notes: Mandibular cortical bone was classified using Klemetti´s Index as normal, moderate, or severely eroded cortex and the trabeculation classified into sparse or normal (mixed or dense). Education included all three levels namely low, medium, and high.

The importance of the covariates for predicting height loss are shown in Table 3. Age is by far the most important parameter. Physical activity was predictive of height loss in some but not all periods, independently of bone structure. Baseline height, smoking and education showed inconsistent associations with height loss in different models, whereas BMI was not significant in any period.

For peer review only

DISCUSSION

Statement of principal findings

The present study was conducted among three systematically recruited birth cohorts from the Prospective Population Study of Women in Gothenburg, Sweden. We found that the longitudinal height loss was significantly predicted by mandibular bone parameters such as severe erosion of the cortex and sparse trabeculation. There was a clear increase in height loss over the follow-up time, in parallel with an increasing cortical erosion and sparseness of trabeculae. Severe cortical erosion was the better risk factor for height loss in the first two periods when the participants were younger, and the trabecular sparseness was a better predictor in the last period corresponding to the oldest ages.

Our findings can be applicable to many settings. Swedish and Norwegian women have the highest frequencies of fragility fractures in the world (27); i.e., they probably have sparser trabeculation and more severe cortical erosion than women in other countries, but if for instance Japanese women have severely eroded cortex (16), they may be of risk for height

For peer review only - http://bmjopen.bmj.com/site/about/guidelines.xhtml

loss as well. Our main finding that severe cortical erosion and sparse trabeculation predict height loss builds on previous work in these cohorts showing mandibular bone structure to be a risk factor for future fractures (24,28,29)

In the first two examination periods, physical activity at the baseline of the study was protective against height loss, which is also in line with other studies (21,30). On the other hand, smoking habits at the baseline had an association only in the last period where the participants were the oldest, in contrast to other studies that have shown strong associations.

Comparisons with other studies

Many previous studies have used height as a proxy for health to compare the welfare of populations, whereas few have investigated how not only height but also height loss could be associated with health in old age (5,10,31). Height is considered a marker of early life health, whereas height loss is a potential risk factor for later life health (5,10,31).

Huang et al. 2013 estimated pre-shrinkage height and studied the association between height loss and different measures of health of older people (10). They found that height loss for both men and women was negatively associated with better education, urban residence, and household economy. Moreover, height loss was positively correlated with poor health outcomes, especially strong for cognition decline (10). Fernihough & McGovern found that height loss among older English men and women across a lifetime decreases by 2 cm to 4 cm on average (31). These number are similar to our findings, where we found an average decrease of 4.3 cm after 37 years.

Jain & Ma 2020 found that mid-aged and older Indonesian women exhibit considerable height loss despite being short to begin with, losing an average of 1.06 cm over 17 years (5). Ethnicity, working in the agricultural sector and availability of local health infrastructure were correlated with loss of height (5). Extreme height loss, greater than 3 cm, was associated with

For peer review only - http://bmjopen.bmj.com/site/about/guidelines.xhtml

8-10 percent lower lung function and grip strength and with an increased mortality (5). They also found that loss of height is higher among older age groups and taller individuals (5), which is in accordance with our results. A recent investigation demonstrated, using national register data linked to the same cohort as described here, that loss of height over 12 years predicted mortality from all-cause and cardiovascular disease (4). This is in line with the study of Jain & Ma (5). Such data is not widely available in prospective epidemiological studies.

As described, height loss is associated with poor health (1,4,5,8-10,30,31). Vertebral discs degenerate far earlier than other musculoskeletal tissues (1) and can lead to back pain and disability in the older adults (1) and in severe cases even vertebral fractures, which have been shown to have direct or indirect consequences such as decreased mobility, depression, hospitalisation, and mortality (22).

Panoramic dental radiography indices, cortical thickness, cortical erosion, and sparse trabeculation, have been suggested as screening tools for identifying risk groups with osteoporosis (16,17,24). Cortical erosion, assessed at dental practices, has been shown to be useful in the identification of undetected low bone mineral density among postmenopausal women (17). However, Okabe et al. 2008 found no significant correlations between cortical erosion assessments and the occurrence of fractures in a group of 80-years old Japanese men and women (15). The difference in the results may be attributed to ethnicity-related anthropometric differences, like height, and the high frequencies of fragility fractures in Swedish and Norwegian women (27). Another difference is that Okabe et al. studied five years of fracture incidence whereas Jonasson et al investigated the same over ten years (15).

Mandibular cortical width is associated with general bone mineral density (16,32),but not with future osteoporotic fractures (28). Sparse trabeculation was shown to be associated with

For peer review only

For peer review only - http://bmjopen.bmj.com/site/about/guidelines.xhtml

a twofold higher risk for future fracture in a younger group and with a three- to fourfold higher risk for future fracture in an older group (29). As far as we know, relationships between height loss and oral indices have not been examined before.

All bone structures undergo an alteration in the bone remodeling mechanism in connection with menopause, leading to greater bone loss than bone formation (33). The imbalance results in trabecular thinning, increase in intra-trabecular spacing, loss of connectivity in the meshwork and intra-cortical erosion, thereby leading to diminished bone strength and increased risk of fracture-(34,35). Taller women have thinner cortical bone (in the appendicular skeleton) and therefore a greater risk for fractures (36). The vertebral bone mass is largely trabecular with only a thin shell of cortical bone. The thin cortex together with the axial compressive load on the vertebra, distributed on fewer and thinner trabeculae, result in an increasing risk for fractures (37,38).

Strengths and limitations

The strengths are the prospective design, the population-based sample, the long follow-up, and the potential applicability of our findings to many settings, since many individuals visit their dentist at least every two years and radiographs are taken. The unique aspect of this study is its design, consisting of long-term panel data on bone structure and height. The main limitations are the fact that height loss reflects various conditions that may make accurate height measures difficult. Moreover, participation rates became lower in the later years, and the drop out for different reasons, mortality included, probably differs between age groups, which might impact the mean height losses and the associations to covariates.

Implications of the findings

In our study population, the sparse trabeculation and the severe cortical erosion of the mandible could be seen to mimic the status of the vertebral trabeculae and intracortical

For peer review only

For peer review only - http://bmjopen.bmj.com/site/about/guidelines.xhtml

erosion of the thin cortical shell of the vertebrae, which would, in turn, explain the potential height reduction. These two bone-related risk factors are important for the development of height loss as well as osteoporosis. A vertebral fracture is often asymptomatic, but it should not be ignored since it is a highly significant risk factor for future fractures. Therefore, using mandible status as a proxy and possible screening tool for the general status of vertebral trabeculae and intracortical bones is interesting for future health promotion.

However, implementation of new methods requires ethical considerations. Prevention has a long tradition in dentistry. Informing patients about their dental hygiene is an integral part of dental practice, whereas screening for or diagnosing osteoporosis/ risk of height loss is not. An ethical dilemma in health promotion is that risk information may have unintended negative consequences for people who are currently free of symptoms. This is demonstrated in a qualitative study of Hvas et al 2005 (39), whose interviews indicated that awareness of osteoporosis risk caused a feeling of worry in some postmenopausal women.

Visual assessments of the mandibular trabecular and cortical bone as conducted in this study are not routine in dental practice today, but simplified screening procedures using artificial intelligence may change this in the future (40). Many postmenopausal women are interested in their health, and if they ask their dentist about the radiographs taken, prudence is needed when the bone is not normal due to sparse trabeculation or severely eroded cortex.

CONCLUSION

Mandibular bone structure alterations such as severe cortical erosion and sparse trabeculation predicted height loss. They may therefore serve as proxy indicators when screening in the early phases of bone degenerative pathogenesis, signaling the ongoing bone remodeling and the need for further clinical attention to older women at risk for height loss.

For peer review only - http://bmjopen.bmj.com/site/about/guidelines.xhtml

Contributors: NNG and LL initiated the study. VS and GJ did the acquisition of data. NNG, LL, VS, GJ and ER contributed to the analysis and interpretation of data. VS and NNG did the statistical analyses. NNG wrote the first draft of the manuscript. NNG, LL, GJ, ER and VS critically revised the manuscript and approved the final version. LL obtained the funding.

Competing interest: None

Funding: This work was supported by grants from the Swedish Research Council for Health, Wing Life and Welfare (Forte, EpiLife 2016-1506), and the Swedish ALF-agreement (ALFGBG 720201).

Data sharing statement: No additional data available.

Ethics Approval: The Prospective Population Study of Women in Gothenburg has been approved by the Regional Ethics Review Board of the University of Gothenburg (*DNR 65-80, 179-92, T453-04*). The examinations comply with the Declaration of Helsinki and informed consent has been obtained from the subjects.

For peer review only

References

1. Hillier TA, Lui LY, Kado DM, LeBlanc ES, Vesco KK, Bauer DC, et al. Height loss in older women: risk of hip fracture and mortality independent of vertebral fractures. J Bone Miner Res 2012;27:153-159. doi: 10.1002/jbmr.558
2. Gavriilidou NN, Pihlsgard M, Elmstahl S. Anthropometric reference data for elderly Swedes and its disease-related pattern. Eur J Clin Nutr 2015;69:1066-1075. doi: 10.1038/ejcn.2015.73.
3. Pluskiewicz W, Adamczyk P, Drozdzowska B. The significance of height loss in postmenopausal women. The results from GO Study. Int J Clin Pract 2021;75:e14009. doi: 10.1111/ijcp.14009.
4. Klingberg S, Mehlig K, Dangol R, Björkelund C, Heitmann BL, Lissner L. Loss of height predicts total and cardiovascular mortality: a cohort study of northern European women. BMJ Open 2021;11:e049122. doi: 10.1136/bmjopen-2021-049122.
5. Jain U, Ma M. Height shrinkage, health and mortality among older adults: Evidence from Indonesia. Econ Hum Biol 2020;37:100863. doi: 10.1016/j.ehb.2020.100863.
6. Perissinotto E, Pisent C, Sergi G, Grigoletto F, Group IW. Anthropometric measurements in the elderly: age and gender differences. Br J Nutr 2002;87:177-186. doi: 10.1079/bjn2001487.
7. Gavriilidou NN, Pihlsgard M, Elmstahl S. High degree of BMI misclassification of malnutrition among Swedish elderly population: Age-adjusted height estimation using knee height and demispan. Eur J Clin Nutr 2015;69:565-571. doi: 10.1038/ejcn.2014.183.
8. Urban JP, Roberts S. Degeneration of the intervertebral disc. Arthritis Res Ther 2003;5:120-130. doi: 10.1186/ar629.

For peer review only

9. Wannamethee SG, Shaper AG, Lennon L, Whincup PH. Height loss in older men: associations with total mortality and incidence of cardiovascular disease. Arch Intern Med 2006;166:2546-2552. doi: 10.1001/archinte.166.22.2546.

10. Huang W, Lei X, Ridder G, Strauss J, Zhao Y. Health, Height, Height Shrinkage, and SES at Older Ages: Evidence from China. Am Econ J Appl Econ 2013;5:86-121. doi: 10.1257/app.5.2.86

11. Kanis JA. Assessment of fracture risk and its application to screening for postmenopausal osteoporosis: synopsis of a WHO report. WHO Study Group. Osteoporos Int 1994;4:368-381. doi: 10.1007/BF01622200

12. Jonasson G, Bankvall G, Kiliaridis S. Estimation of skeletal bone mineral density by means of the trabecular pattern of the alveolar bone, its interdental thickness, and the bone mass of the mandible. Oral Surg Oral Med Oral Pathol Oral Radiol Endod 2001;92:346-352. doi: 10.1067/moe.2001.116494.

13. Lindh C, Horner K, Jonasson G, Olsson P, Rohlin M, Jacobs R, et al. The use of visual assessment of dental radiographs for identifying women at risk of having osteoporosis: the OSTEODENT project. Oral Surg Oral Med Oral Pathol Oral Radiol Endod 2008;106:285-293. doi: 10.1016/j.tripleo.2007.09.008.

14. Lindh C, Petersson A, Rohlin M. Assessment of the trabecular pattern before endosseous implant treatment: diagnostic outcome of periapical radiography in the mandible. Oral Surg Oral Med Oral Pathol Oral Radiol Endod 1996;82:335-243. doi: 10.1016/s1079-2104(96)80363-5

15. Okabe S, Morimoto Y, Ansai T, Yoshioka I, Tanaka T, Taguchi A, et al. Assessment of the relationship between the mandibular cortex on panoramic radiographs and the risk of bone fracture and vascular disease in 80-year-olds. Oral Surg Oral Med Oral Pathol Oral Radiol Endod 2008;106:433-424. doi: 10.1016/j.tripleo.2007.09.013.

For peer review only - http://bmjopen.bmj.com/site/about/guidelines.xhtml

16. Taguchi A. Panoramic radiographs for identifying individuals with undetected osteoporosis. Japanese Dental Science Review 2009;45:109-120. doi.org/10.1016/j.jdsr.2009.05.001

17. Taguchi A, Ohtsuka M, Nakamoto T, Naito K, Tsuda M, Kudo Y, et al. Identification of post-menopausal women at risk of osteoporosis by trained general dental practitioners using panoramic radiographs. Dentomaxillofac Radiol 2007;36:149-154. doi: 10.1259/dmfr/31116116.

18. Lissner L, Skoog I, Andersson K, Beckman N, Sundh V, Waern M, et al. Participation bias in longitudinal studies: experience from the Population Study of Women in Gothenburg, Sweden. Scand J Prim Health Care 2003;21:242-247. doi: 10.1080/02813430310003309-1693.

19. Bengtsson C, Ahlqwist M, Andersson K, Bjorkelund C, Lissner L, Soderstrom M. The Prospective Population Study of Women in Gothenburg, Sweden, 1968-69 to 1992-93. A 24-year follow-up study with special reference to participation, representativeness, and mortality. Scand J Prim Health Care 1997;15:214-219. doi: 10.3109/02813439709035031.

20. Klemetti E, Kolmakov S, Kroger H. Pantomography in assessment of the osteoporosis risk group. Scand J Dent Res 1994;102:68-72. doi: 10.1111/j.1600-0722.1994.tb01156.x.

21. Sagiv M, Vogelaere PP, Soudry M, Ehrsam R. Role of physical activity training in attenuation of height loss through aging. Gerontology 2000;46:266-270. doi: 10.1159/000022170.

22. Xu W, Perera S, Medich D, Fiorito G, Wagner J, Berger LK, et al. Height loss, vertebral fractures, and the misclassification of osteoporosis. Bone 2011;48:307-311. doi: 10.1016/j.bone.2010.09.027.

For peer review only - http://bmjopen.bmj.com/site/about/guidelines.xhtml

23. Thornton MJ, Sedlak CA, Doheny MO. Height change and bone mineral density: revisited. Orthop Nurs 2004;23:315-320. doi: 10.1097/00006416-200409000-00007.

24. Jonasson G, Sundh V, Ahlqwist M, Hakeberg M, Bjorkelund C, Lissner L. A prospective study of mandibular trabecular bone to predict fracture incidence in women: a low-cost screening tool in the dental clinic. Bone 2011;49:873-879. doi: 10.1016/j.bone.2011.06.036.

25. Grimby G, Börjesson M, Jonsdottir IH, Schnohr P, Thelle DS, Saltin B. The "Saltin-Grimby Physical Activity Level Scale" and its application to health research. Scand J Med Sci Sports 2015;25 Suppl 4:119-25. doi: 10.1111/sms.12611

26. Kaufman JS, Cooper RS. Seeking Causal Explanations in Social Epidemiology. Am J Epidemiol 1999;150:113-120. doi: 10.1093/oxfordjournals.aje.a009969.

27. SBU. Osteoporos – prevention, diagnostik och behandling. En systematisk litteraturöversikt. Stockholm.: Statens beredning för medicinsk utvärdering (SBU). 2003.

28. Jonasson GB, Sundh V, Hakeberg M, Ahlqwist M, Lissner L, Hange D. Evaluation of clinical and radiographic indices as predictors of osteoporotic fractures: a 10-year longitudinal study. Oral Surg Oral Med Oral Pathol Oral Radiol 2018;125:487-494. doi: 10.1016/j.oooo.2017.11.009.

29. Sundh V, Hange D, Ahlqwist M, Hakeberg M, Lissner L, Jonasson G. FRAX and mandibular sparse trabeculation as fracture predictors: a longitudinal study from 1980 to 2002. Eur J Oral Sci 2017;125:135-140. doi: 10.1111/eos.12341.

30. Mai X, Marshall B, Hovey KM, Sperrazza J, Wactawski-Wende J. Risk factors for 5-year prospective height loss among postmenopausal women. Menopause 2018;25:883-889. doi: 10.1097/GME.0000000000001108.

For peer review only

31. Fernihough A, McGovern ME. Physical Stature Decline and the Health Status of the Elderly Population in England. Econ Hum Biol 2015;16:30-44. doi:10.1016/j.ehb.2013.12.010.

32. Devlin H, Horner K. Mandibular radiomorphometric indices in the diagnosis of reduced skeletal bone mineral density. Osteoporos Int 2002;13:373-378. doi: 10.1007/s001980200042.

33. Lerner UH. Bone remodeling in post-menopausal osteoporosis. J Dent Res 2006;85:584-595. doi: 10.1177/154405910608500703.

34. Bala Y, Zebaze R, Seeman E. Role of cortical bone in bone fragility. Curr Opin Rheumatol 2015;27(4):406-13. doi: 10.1097/BOR.0000000000000183.

35. Bjornerem A, Wang X, Bui M, Ghasem-Zadeh A, Hopper JL, Zebaze R, et al. Menopause-Related Appendicular Bone Loss is Mainly Cortical and Results in Increased Cortical Porosity. J Bone Miner Res 2018;33:598-605. doi: 10.1002/jbmr.3333.

36. Bjornerem A, Bui QM, Ghasem-Zadeh A, Hopper JL, Zebaze R, Seeman E. Fracture risk and height: an association partly accounted for by cortical porosity of relatively thinner cortices. J Bone Miner Res 2013;28:2017-2026. doi: 10.1002/jbmr.1934.

37. Adams MA, Hutton WC. The Mechanical Function of the Lumbar Apophyseal Joints. Spine 1983;8. doi: 10.1097/00007632-198304000-00017.

38. Giambini H, Salman Roghani R, Thoreson AR, Melton LJ, 3rd, An K-N, Gay RE. Lumbar trabecular bone mineral density distribution in patients with and without vertebral fractures: a case-control study. Eur Spine J 2014;23:1346-1353. doi: 10.1007/s00586-014-3205-2.

For peer review only - http://bmjopen.bmj.com/site/about/guidelines.xhtml

39. Hvas L, Reventlow S, Jensen HL, Malterud K. Awareness of risk of osteoporosis may cause uncertainty and worry in menopausal women. Scand J Public Health 2005;33:203–207. doi: 10.1080/14034940510005716

40. Jonasson G, Skoglund I, Rythén M. The rise and fall of the alveolar process: Dependency of teeth and metabolic aspects. Arch Oral Biol 2018;96:195-200. https://doi.org/10.1016/j.archoralbio.2018.09.016

For peer review only

For peer review only - http://bmjopen.bmj.com/site/about/guidelines.xhtml

Figure legend

Figure 1: Flow chart.

For peer review only

For peer review only - http://bmjopen.bmj.com/site/about/guidelines.xhtml

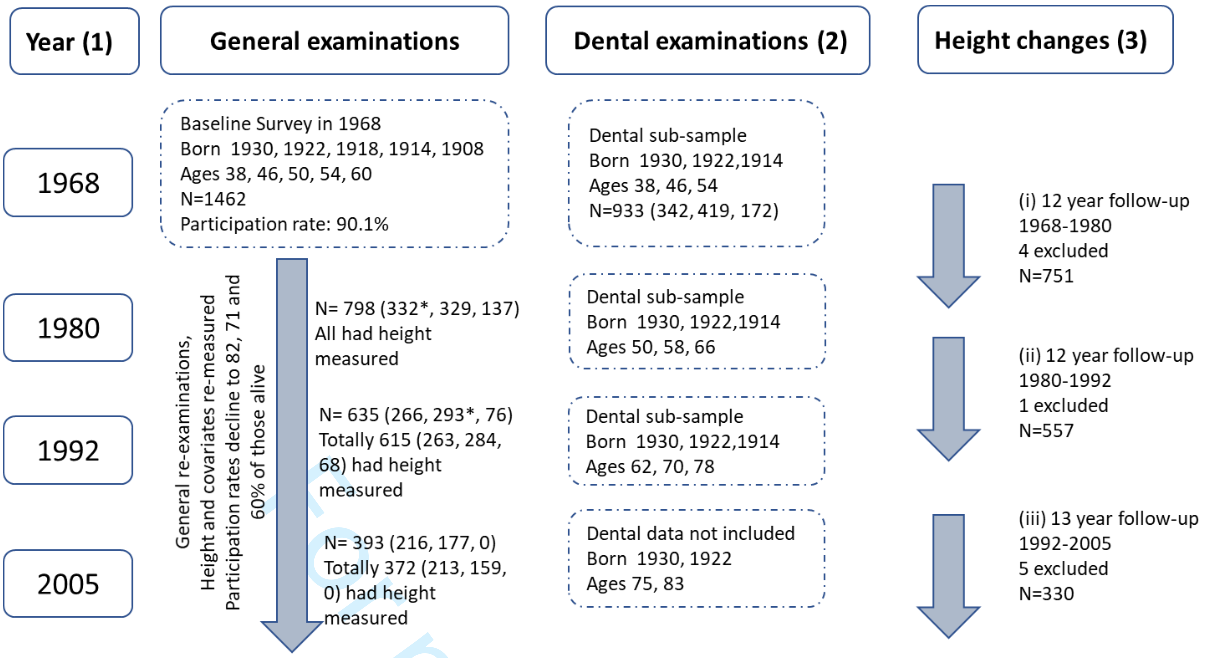

(1) Each examination period starting in September and ending in June of following year, i.e., 1968-69, 1980-81, 1992-93, 2005-06
(2) Dental examination conducted shortly after general examination in three of original age groups. Mandibular bone structure (cortical and trabecular bone) is the main independent variable
(3) Heights were measured at general examinations, and the change in height was estimated at 12- or 13-year intervals, after excluding any increases of over 2 cm

For peer review only

STROBE 2007 (v4) Statement—Checklist of items that should be included in reports of *cohort studies*

Section/Topic	Item #	Recommendation	Reported on page #
**Title and abstract**	1	(*a*) Indicate the study's design with a commonly used term in the title or the abstract	1 and 2
		(*b*) Provide in the abstract an informative and balanced summary of what was done and what was found	2
**Introduction**			
Background/rationale	2	Explain the scientific background and rationale for the investigation being reported	5
Objectives	3	State specific objectives, including any prespecified hypotheses	6
**Methods**			
Study design	4	Present key elements of study design early in the paper	2-5
Setting	5	Describe the setting, locations, and relevant dates, including periods of recruitment, exposure, follow-up, and data collection	2, 5
Participants	6	(*a*) Give the eligibility criteria, and the sources and methods of selection of participants. Describe methods of follow-up	5
		(*b*) For matched studies, give matching criteria and number of exposed and unexposed	-
Variables	7	Clearly define all outcomes, exposures, predictors, potential confounders, and effect modifiers. Give diagnostic criteria, if applicable	7, 8
Data sources/ measurement	8*	For each variable of interest, give sources of data and details of methods of assessment (measurement). Describe comparability of assessment methods if there is more than one group	*7, 8*
Bias	9	Describe any efforts to address potential sources of bias	11, 12
Study size	10	Explain how the study size was arrived at	7
Quantitative variables	11	Explain how quantitative variables were handled in the analyses. If applicable, describe which groupings were chosen and why	8
Statistical methods	12	(*a*) Describe all statistical methods, including those used to control for confounding	8
		(*b*) Describe any methods used to examine subgroups and interactions	8, 10
		(*c*) Explain how missing data were addressed	6
		(*d*) If applicable, explain how loss to follow-up was addressed	-
		(*e*) Describe any sensitivity analyses	-
**Results**			

For peer review only

Participants	13*	(a) Report numbers of individuals at each stage of study—eg numbers potentially eligible, examined for eligibility, confirmed eligible, included in the study, completing follow-up, and analysed	6, 7 +fig 1
		(b) Give reasons for non-participation at each stage	6, 7
		(c) Consider use of a flow diagram	Fig 1
Descriptive data	14*	(a) Give characteristics of study participants (eg demographic, clinical, social) and information on exposures and potential confounders	6, 7
		(b) Indicate number of participants with missing data for each variable of interest	Table 1
		(c) Summarise follow-up time (eg, average and total amount)	Fig 1 + tables
Outcome data	15*	Report numbers of outcome events or summary measures over time	5-8
Main results	16	(*a*) Give unadjusted estimates and, if applicable, confounder-adjusted estimates and their precision (eg, 95% confidence interval). Make clear which confounders were adjusted for and why they were included	Tables
		(*b*) Report category boundaries when continuous variables were categorized	Tables
		(*c*) If relevant, consider translating estimates of relative risk into absolute risk for a meaningful time period	-
Other analyses	17	Report other analyses done—eg analyses of subgroups and interactions, and sensitivity analyses	-
**Discussion**			
Key results	18	Summarise key results with reference to study objectives	10
**Limitations**			
Interpretation	20	Give a cautious overall interpretation of results considering objectives, limitations, multiplicity of analyses, results from similar studies, and other relevant evidence	14
Generalisability	21	Discuss the generalisability (external validity) of the study results	2, 11
**Other information**			
Funding	22	Give the source of funding and the role of the funders for the present study and, if applicable, for the original study on which the present article is based	15

*Give information separately for cases and controls in case-control studies and, if applicable, for exposed and unexposed groups in cohort and cross-sectional studies.

Note: An Explanation and Elaboration article discusses each checklist item and gives methodological background and published examples of transparent reporting. The STROBE checklist is best used in conjunction with this article (freely available on the Web sites of PLoS Medicine at http://www.plosmedicine.org/, Annals of Internal Medicine at http://www.annals.org/, and Epidemiology at http://www.epidem.com/). Information on the STROBE Initiative is available at www.strobe-statement.org.

For peer review only

BMJ Open

Does mandibular bone structure predict subsequent height loss? A longitudinal cohort study of women in Gothenburg, Sweden

Journal:	*BMJ Open*
Manuscript ID	bmjopen-2022-066844.R3
Article Type:	Original research
Date Submitted by the Author:	20-Apr-2023
Complete List of Authors:	Gavriilidou, Nivetha N; Karolinska Institute, Department of Dental Medicine<br>Jonasson, Grethe; University of Gothenburg, Department of Behavioral and Community Dentistry<br>Sundh, Valter; University of Gothenburg, School of Public Health and Community Medicine, Institute of Medicine, Sahlgrenska Academy,<br>Rothenberg, Elisabet ; Kristianstad University, Department of Nursing and Integrated Health Sciences<br>Lissner, Lauren; University of Gothenburg Sahlgrenska Academy, School of Public Health and Community Medicine, Institute of Medicine, Sahlgrenska Academy,
**Primary Subject Heading**:	Dentistry and oral medicine
Secondary Subject Heading:	Dentistry and oral medicine
Keywords:	ORAL MEDICINE, Bone diseases < ORTHOPAEDIC & TRAUMA SURGERY, PUBLIC HEALTH

SCHOLARONE™
Manuscripts

I, the Submitting Author has the right to grant and does grant on behalf of all authors of the Work (as defined in the below author licence), an exclusive licence and/or a non-exclusive licence for contributions from authors who are: i) UK Crown employees; ii) where BMJ has agreed a CC-BY licence shall apply, and/or iii) in accordance with the terms applicable for US Federal Government officers or employees acting as part of their official duties; on a worldwide, perpetual, irrevocable, royalty-free basis to BMJ Publishing Group Ltd ("BMJ") its licensees and where the relevant Journal is co-owned by BMJ to the co-owners of the Journal, to publish the Work in this journal and any other BMJ products and to exploit all rights, as set out in our licence.

The Submitting Author accepts and understands that any supply made under these terms is made by BMJ to the Submitting Author unless you are acting as an employee on behalf of your employer or a postgraduate student of an affiliated institution which is paying any applicable article publishing charge ("APC") for Open Access articles. Where the Submitting Author wishes to make the Work available on an Open Access basis (and intends to pay the relevant APC), the terms of reuse of such Open Access shall be governed by a Creative Commons licence – details of these licences and which Creative Commons licence will apply to this Work are set out in our licence referred to above.

Other than as permitted in any relevant BMJ Author's Self Archiving Policies, I confirm this Work has not been accepted for publication elsewhere, is not being considered for publication elsewhere and does not duplicate material already published. I confirm all authors consent to publication of this Work and authorise the granting of this licence.

For peer review only

Does mandibular bone structure predict subsequent height loss? A longitudinal cohort study of women in Gothenburg, Sweden

Nivetha N Gavriilidou DDS MPH PhD, [a] Grethe Jonasson DDS PhD, [b] Valter Sundh BSc, [c] Elisabet Rothenberg RD PhD, [d] Lauren Lissner MPH PhD. [e]

[a] Karolinska Institute, Department of Dental Medicine, Institute of Odontology, Karolinska Institute, Sweden. Email: nivetha.gavriilidou@ki.se

[b] Department of Behavioral and Community Dentistry, Institute of Odontology at the Sahlgrenska Academy, University of Gothenburg, Sweden. Email: grethe.jonasson@gu.se

[c.] School of Public Health and Community Medicine, Institute of Medicine, Sahlgrenska Academy, University of Gothenburg, Sweden. Email: valter.sundh@gu.se

[d] Department of Nursing and Integrated Health Sciences, Faculty of Health Sciences, Kristianstad University, Sweden. Email: elisabet.rothenberg@hkr.se

[e] School of Public Health and Community Medicine, Institute of Medicine, Sahlgrenska Academy, University of Gothenburg, Sweden. Email: lauren.lissner@gu.se

Corresponding author: Grethe Jonasson, Department of Behavioral and Community Dentistry, Institute of Odontology at the Sahlgrenska Academy, University of Gothenburg, Sweden. Email: grethe.jonasson@gu.se; tel +46 709285671

Short title: Mandibular bone structure and stature shrinkage.

For peer review only - http://bmjopen.bmj.com/site/about/guidelines.xhtml

ABSTRACT

Background Several risk factors for loss of height with increasing age have been identified.

Objective To investigate if mandibular bone structure predicts future height loss in middle-aged and elderly Swedish women.

Design Prospective cohort study with longitudinally measured heights, radiographical assessments of the cortical bone using Klemetti´s Index (normal, moderate, or severely eroded cortex), and classification of the trabecular bone using an index proposed by Lindh et al. (sparse, mixed, or dense trabeculation). No intervention was performed.

Setting Gothenburg, Sweden.

Participants A population-based sample of 937 Swedish women born in 1914, 1922 and 1930 was recruited. At the baseline examination, the ages were 38, 46, and 54. All had undergone a dental examination with panoramic radiographs of the mandible, and a general examination including height measurements on at least 2 occasions.

Main outcome measure Height loss was calculated over three 12-13-year periods (1968-80, 1980-92, 1992- 2005).

Main results. Mean annual height loss measures were 0.075cm/year, 0.08 cm/year, and 0.18 cm/year over the 3 observation intervals, corresponding to absolute decreases of 0.9 cm, 1.0 cm, and 2.4 cm. Cortical erosion in 1968, 1980 and 1992 significantly predicted height loss 12 years later. Sparse trabeculation in 1968, 1980 and 1992 also predicted significant shrinkage over 12 or 13 years. Multivariable regression analyses adjusting for baseline covariates such as height, birth year, physical activity, smoking, body mass index, and education yielded consistent findings except for cortical erosion 1968-80.

For peer review only

For peer review only - http://bmjopen.bmj.com/site/about/guidelines.xhtml

Conclusion Mandibular bone structure characteristics such as severe cortical erosion and sparse trabeculation may serve as early risk factors for height loss. Since most individuals visit their dentist at least every two years and radiographs are taken, a collaboration between dentists and physicians may open opportunities for predicting future risk of height loss.

Keywords Ageing, body height, cortical bone, dental radiography, mandible, stature decline, trabecular bone, women's health.

For peer review only

For peer review only - http://bmjopen.bmj.com/site/about/guidelines.xhtml

Strengths and limitations of this study

- The strengths are the prospective design, the population-based sample, the long follow-up, repeated measurements of height for the same individuals, and that our findings can be applicable to many settings.
- The main limitation is that loss of height could reflect different conditions which may make accurate height estimates difficult.
- The participation rates became lower in the later years, and the drop out for different reasons, mortality included, probably differs between age groups, which might impact the mean height losses.

For peer review only

INTRODUCTION

Height loss in older women is incrementally more pronounced at ages over 75 years. It has been found to be associated with increased morbidity and mortality (1-5). Several causes have been identified that lead to loss of height among older adults. Height loss could indicate age-associated progressive skeletal deformation such as kyphoscoliosis, flattening of plantar arch and altered posture (6,7), and/or degenerative processes including osteoporosis, vertebral compression, attrition of intervertebral discs, and vertebral fractures (8). These alterations make accurate height estimation a challenge. Comorbidities of major height loss in older adults include functional impairments such as dependence in daily life activities, cognitive impairment, and increased overall mortality risk (1,5). Height loss over 5cm has been shown to predict non-spinal fractures such as hip fractures and mortality independent of vertebral fractures (1,6,7,9,10). Height loss in women exceeding 4 cm has been found to be related to fracture probability of over 50% (3). In men, health consequences of height loss over 3 cm include increased risk of all-cause mortality and coronary heart disease (9).

Osteoporosis is a widely acknowledged risk factor for future fractures in the elderly. According to the World Health Organization, it is defined as bone density lower than 2.5 standard deviations under that of young women using the bone mineral density of the hip with dual X-ray absorptiometry (DXA) (11). Simple, practical, and relevant techniques are needed for early identification of the onset or extent of skeletal degeneration. Therefore, researchers have used oral variables such as optical density (12), sparse trabeculation (12-14), cortical width (15), and cortical erosion (15-17) for the prediction of osteoporosis and fracture.

Intraoral radiographs are usually an integral part of the routine oral examination. Assessment of mandibular structure, the cortical and trabecular bone pattern may be studied through visual inspection, hence providing a simple screening method for the evaluation of the bone condition. The hypothesis of the present study is that the mandibular bone contains

For peer review only

For peer review only - http://bmjopen.bmj.com/site/about/guidelines.xhtml

sufficient information about the general bone condition to predict future height loss. The objective is to investigate two proxies for general bone mineral density, namely mandibular cortical erosion, and trabecular coarseness, as predictors of height loss over subsequent periods of 12-13 years.

METHODS

Study population

The Prospective Population Study of Women in Gothenburg, Sweden, is an ongoing longitudinal study initiated in 1968 in a cohort of women aged 38, 46, 50, 54, or 60 years. At that time, a representative sample of women in Gothenburg was identified using the Revenue Office register with respect to the dates of birth. From the full baseline cohort, those born in 1914, 1922, and 1930 were included in the present analytic sample (flowchart, Figure 1). The original survey included general health and dental examinations, which were repeated over the years. Each wave of survey was started in the fall and completed in the spring of the following year, i.e., 1968-69, 1980-81, 1992-93, and 2004-05, and is hereafter referred to by its starting year. The participation rate in the initial examination was over 90% (18,19). A non-participation analysis of the 24-year follow-up in 1992 showed that the women who declined to participate in the first survey did not differ significantly from participants except for the long-term survival, which was lower in the initial refusers. The sample remained representative of the general population in terms of parameters such as blood pressure, weight, height, BMI, waist and hip circumference, serum cholesterol, triglyceride, and urate (19).

Inclusion criteria

In total, 937 women born in 1914, 1922 or 1930, were included in the present analysis. The inclusion criteria were availability of at least one panoramic radiograph in 1968, 1980, or

For peer review only

1992 from which it was possible to assess the mandibular cortex erosion and sparsity of trabeculae, plus availability of a baseline and at least one subsequent height measure. In the first period (1968-1989), four individuals were excluded due to an *increase* in height of at least 2 cm. Number excluded 1980-1992 was one, and it was five in the period 1992-2005. Results from previous observational studies using the same cohorts have shown that the number of participants should be sufficient for studying common health endpoints.

Patient and Public Involvement

No patients involved.

Main outcome

The main outcome variable was height loss during the 12-13 years following the dental examination. At all examinations, height and weight were assessed by standard anthropometric measurement techniques. A stadiometer was used to measure the participants' body heights (without shoes). Measurement of height was generally performed early in the day. Height loss was calculated as baseline height minus height at follow-up. Thus, a positive value indicated height loss.

Height change was assessed from follow-up examinations 12-13 years later, until 2005. The exclusion criterion was an observed *increase* in height of at least 2 cm during any of these follow-up periods, attributable to probable measurement errors.

There were three 12–13-year follow-up periods namely 1968-1980, 1980-1992, and 1992-2005, where the first period comprised middle-aged women initially aged 38, 46, 54 years, the second period with a subset of these women from ages 62, 70 and 78 years, and the third and the latest period starting at ages 75 and 83 years (no follow-up from 1914 birth cohort) (Figure 1).

Bone structure and other covariates

For peer review only - http://bmjopen.bmj.com/site/about/guidelines.xhtml

Radiographic assessment of mandibular bone structures was included as part of the dental examination. In the present analysis, the main independent variables were two components of mandibular bone status. On visual inspection of the panoramic radiograph of a mandible, the cortical bone was categorised using Klemetti´s Index as mild/none (normal cortex), moderate, or severely eroded cortex (20). A normal cortex means an even endosteal margin; a moderate eroded cortex has semilunar defects in the endosteal margin, whereas a severely eroded cortex has heavy endosteal cortical porosities. The variable was dichotomised into "*severely eroded cortex"* (1) and "*non-severely eroded"* (0). The second component, the trabeculation, was classified using an index proposed by Lindh et al. 1996 as sparse, mixed, or dense (13,14). Sparse trabeculation means large intertrabecular spaces in the premolar area. Dense trabeculation has small intertrabecular spaces. Mixed dense and sparse trabeculation is mostly dense at the crest and sparse apically. The variable was dichotomised into "*sparse" trabeculation* (1) and "*normal" (mixed and dense) trabeculation* (0).

Baseline variables such as height, body mass index (BMI), smoking, physical activity, and education, were considered potential covariates that could be associated with mandibular erosion and trabecular pattern, as well as height loss (21-24). The survey questionnaire regarding smoking habit had 6 possible responses: Never smoked (0), Previous smoker but quit in the last 15 years (1), Previous smoker but quit since last year (2), Quit smoking since last year (3), Current smoker without deep inhalation of the smoke (4), and Current smoker with deep inhalation of the smoke (5). This variable was dichotomised to "*No smoking*" (0) including response options 0, 1, 2, and "*Smoking*" (1) including options 3, 4, or 5. The variable physical activity was categorised as (i) almost no physical activity or none (sedentary), (ii) at least 4 hours/ a week of physical activity, (iii) regular physical activity and (iiii) regular intense training and competitions (25). This variable was dichotomised as: "*physically inactive*" (0) including the first response and "*physically active*" (1) including the

For peer review only - http://bmjopen.bmj.com/site/about/guidelines.xhtml

latter three choices. Education was considered a proxy for socioeconomic status as proposed by Kaufmann (26) and categorised as low: pre-high school (up to 9 years); medium: high school with ≤2 years at university; and high: 3 or more years at university. The variable was dichotomized into "*low education*" (1) versus "*higher*" (0).

Statistical analyses

Mann-Whitney Two-Sample Test was used for differences between two groups. In order to test the importance of lifestyle factors, linear multiple regression analyses were performed, where height loss was the dependent variable. The following variables were tested for independent prediction of height loss: the two dichotomous mandibular bone variables, smoking, physical activity, height, body mass index (BMI), education and age (birth year). Height, height loss and BMI were continuous variables, whereas the two bone variables, smoking, physical activity, and education were dichotomised as described above. The significance level was $p<0.05$). Statistical analyses were performed with SAS version 9.2 software (SAS Institute Inc., Cary, NC).

RESULTS

Selected characteristics of the sample are shown in Table 1. Mean age at the four height measurements (year 1968, 1980, 1992, and 2005) were 44.9, 56.9, 68.0, and 78.8 years respectively, corresponding to three periods of height change (1968-80, 1980-92, and 1992-2005). There was a rising trend in the proportion of women with severe cortical erosion from 3.2% among the participants in the period 1968-1980, to 11.1% in the period 1980-1992 and up to 49.8% in the period 1992-2005. Similarly, prevalence of sparse trabeculation increased from 20.6% among the participants 1968-1980, to 33.5% in 1980-1992 and up to 41.6% in the period 1992-2005. At the same time, the mean height loss over the three survey periods was

For peer review only - http://bmjopen.bmj.com/site/about/guidelines.xhtml

For peer review only

0.9 cm, 1.0 cm, and 2.4 cm, with corresponding annual decreases of 0.075, 0.08, and 0.18 cm/year (Table 1).

Table 1: Descriptive characteristics of the population sub-samples born in 1914, 1922 and 1930 at examination year 1968 followed up across periods 1968-80, 1980-92, 1992-2005.

	1968	1980	1992	2005
Age of women born 1930 years (N)	38 (342)	50 (289)	62 (251)	75 (190)
Born 1922	46 (419)	58 (327)	70 (239)	83 (140)
Born 1914	54 (172)	66 (135)	78 (67)	91 (0) [a]
	Total (933)	Total (751)	Total (557)	Total (330)
**Parameters at the end of the period**	**1968**	**1968-80**	**1980-92**	**1992-2005**
Mean age (SD)	44.9 (5.8)	56.9 (5.8)	68.0 (5.4)	78.8 (3.8)
Mean height in cm (SD)	163.9 (6.0)	163.0 (6.1)	162.1 (6.2)	160.4 (6.1)
Almost no physical activity N (%)	157 (16.8)	221 (29.4)	117 (21.0)	25 (9.3)
Active Smokers N (%)	308 (41.0)	257 (34.3)	115 (20.6)	30 (9.2)
Education level [b]				
Low	630 (67.7)	506 (67.4)	364 (65.4)	209 (63.3)
Medium	251 (27.0)	205 (27.3)	159 (28.5)	93 (28.2)
High	49 (5.3)	39 (5.2)	33 (5.9)	28 (8.5)
Mean body mass index (kg/m^2)	23.7 (3.7)	24.7 (3.9)	26.4 (4.2)	26.2 (4.5)
Weight (kg)	63.6 (10.8)	66.0 (11.2)	69.0 (11.9)	68.8 (11.8)
**Period 1968- 80**				
Mean height Loss (cm (SD))	0.90 (0.89)			
Mean annual height loss (cm/year)	0.075			
**Period 1980- 92**				
Mean height Loss (cm (SD))		1.0 (1.21)		
Mean annual height loss (cm/year)		0.08		
**Period 1992- 2005**				
Mean height Loss (cm (SD))			2.4 (1.54)	
Mean annual height loss (cm/year)			0.18	
**Mandibular parameters at the start of the period**				
Cortical Erosion *N (%)*				
Mild/None	583 (65.2)	451 (62.9)	175 (33.6)	5 (8.2)
Moderate	283 (31.7)	243 (33.9)	288 (55.3)	128 (42,0)
Severely eroded	28 (3.1)	23 (3.2)	58 (11.1)	152(49.8)
Trabeculation *N (%)*				
Dense	236 (28.8)	133 (20.3)	78 (14.8)	15 (9.3)
Mixed	414 (50.6)	302 (46.2)	208 (39.5)	79 (49.1)
Sparse	169 (20.6)	219 (33.5)	241 (45.7))	67 (41.6)

Notes: Cortical bone was classified using Klemetti´s Index as mild, moderate, or severe erosion and the trabeculation classified into sparse, mixed, or dense. Number excluded based on shrinkage values over period 1968-1980: 4, 1980-1992: 1, 1968-1992: 4, 1992-2005: 5.

[a]): There were none in this age group in the follow up period 1992-2005.

Not all participants with height measurements had an assessment of cortical erosion and even fewer had trabecular assessment due to edentulism.

[b]): Education level categories: Low: pre-high school level (up to 9 years), Medium: high school level with ≤2 years at university, High: 3 or more years at university.

For peer review only - http://bmjopen.bmj.com/site/about/guidelines.xhtml

Table 2 describes prospective analyses of association between mandibular bone structure at the three age periods and subsequent height loss, without covariate adjustment.

Table 2: Height loss in relation to cortical erosion and trabecular spacing.

**Mandibular bone structure**	**Height loss (1968-80) [a] N= 751**			**Height loss (1980-92) [a)] N= 557**			**Height loss (1992-2005) [b)] N= 330**		
**Cortical Erosion**	Mean±SD	Rate/ year	p	Mean±SD	Rate/ year	p	Mean±SD	Rate/ year	p
Normal	0.89±0.88	0.07 0.12	0.002	0.90±1.29	0.08 0.14	<0.001	2.10±1.76	0.16 0.20	0.034
Severe	1.46±0.75			1.66±1.15			2.56±1.77		
**Trabecular bone**									
Normal	0.88±0.86	0.07 0.08	0.011	0.81±1.04	0.07 0.09	0.004	2.16±1.26	0.16 0.20	0.002
Sparse	1.01±0.99			1.11±1.15			2.72±1.82		

Cortical erosion was dichotomized into normal (mild or moderate) erosion and severe erosion. The trabeculation was classified into normal (mixed or dense) or sparse.

For peer review only

In all periods height loss was highest in the groups with severe cortical erosion and the groups with sparse trabeculation (Table 2). The difference in shrinkage between the group with normal versus severe erosion was 0.57 cm in the early period, 0.76 cm in the middle period, and 0.46 cm in the last period (Table 2). The difference in height loss between the group with normal/mixed and that with sparse trabeculation was 0.13 cm in the early period, 0.30 cm in the middle period, and 0.56 cm in the last period. Multivariable linear regression analyses in Table 3 confirmed the significant associations between the height loss and cortical erosion during the periods 1980-92 and 1992-2005 but not 1968-80, after adjusting for covariates as height, age (birth year), physical activity, smoking, education, and BMI. The trabeculation showed a significant correlation with height loss in all three periods (Table 3).

For peer review only - http://bmjopen.bmj.com/site/about/guidelines.xhtml

Table 3: Linear multivariable regression models predicting height loss over time intervals 1968-1980, 1980-1992 and 1992-2005. The prediction is based on cortical erosion and trabeculation (both dichotomous) adjusting for covariates height, year of birth, physical activity, smoking, education, and body mass index (BMI).

	**Height loss (1968-80) N= 751**		**Height loss (1980-92) N= 557**		**Height loss (1992-2005) N=330**	
	Coefficient (Std Error)	P value	Coefficient Std Error	P value	Coefficient Std Error	P value
**Severe Erosion**	0.20 (0.18)	0.27	0.48 (0.19)	0.011	0.43 (0.23)	0.050
Height at baseline	0.01 (0.01)	0.22	0.02 (0.01)	0.051	0.03 (0.02)	0.098
Birth year	-0.05 (0.01)	<0.001	-0.06 (0.01)	<0.001	-0.02 (0.03)	0.551
Physical activity	-0.11 (0.06)	0.07	-0.13 (0.10)	0.094	-0.019 (0.17)	0.907
Smoking	-0.02 (0.01)	0.27	0.06 (0.03)	0.031	0.120 (0.07)	0.072
Education	0.02 (0.07)	0.81	0.15 (0.12)	0.218	0.25 (0.23)	0.273
BMI	0.01 (0.01)	0.34	0.001 (0.02)	0.929	-0.015 (0.03)	0.609
**Sparse Trabeculation**	0.16 (0.08)	0.038	0.24 (0.11)	0.023	0.45 (0.22)	0.040
Height at baseline	0.01 (0.01)	0.056	0.02 (0.01)	0.021	0.03 (0.02)	0.131
Birth year	-0.06 (0.01)	<0.001	-0.06 (0.01)	<0.001	-0.02 (0.03)	0.450
Physical activity	-0.14 (0.07)	0.019	-0.12 (0.07)	0.106	-0.10 (0.16)	0.528
Smoking	-0.02 (0.02)	0.320	0.018 (0.02)	0.439	0.13 (0.05)	0.011
Education	-0.02 (0.07)	0.773	0.14 (0.11)	0.190	0.20 (0.23)	0.388
BMI	0.012 (0.01)	0.239	0.02 (0.02)	0.165	-0.01 (0.03	0.717

Notes: Cortical erosion was dichotomized into severe erosion and normal (mild or moderate) erosion. The trabeculation was classified into sparse or normal (mixed or dense).

The importance of the covariates for predicting height loss are shown in Table 3. Age is by far the most important parameter. Physical activity, baseline height, and smoking showed inconsistent associations with height loss in different models, whereas BMI and education were not significant in any period.

DISCUSSION

Statement of principal findings

The present study was conducted among three systematically recruited birth cohorts from the Prospective Population Study of Women in Gothenburg, Sweden. We found that the

For peer review only - http://bmjopen.bmj.com/site/about/guidelines.xhtml

longitudinal height loss was significantly predicted by mandibular bone parameters such as severe erosion of the cortex and sparse trabeculation. There was a clear increase in height loss over the follow-up time, in parallel with aging and an increasing cortical erosion and sparseness of trabeculae.

Our findings can be applicable to many settings. Swedish and Norwegian women have the highest frequencies of fragility fractures in the world (27); i.e., they probably have sparser trabeculation and more severe cortical erosion than women in other countries, but if for instance Japanese women have severely eroded cortex (16), they may be of risk for height loss as well. Our main finding that severe cortical erosion and sparse trabeculation predict height loss builds on previous work in these cohorts showing mandibular bone structure to be a risk factor for future fractures (24,28,29)

Comparisons with other studies

Many previous studies have used height as a proxy for health to compare the welfare of populations, whereas few have investigated how not only height but also height loss could be associated with health in old age (5,10,30,31). Height is considered a marker of early life health, whereas height loss is a potential risk factor for later life health (5,10,30,31).

Huang et al. 2013 estimated pre-shrinkage height and studied the association between height loss and different measures of health of older people (10). They found that height loss for both men and women was negatively associated with better education, urban residence, and household economy. Moreover, height loss was positively correlated with poor health outcomes, especially strong for cognition decline (10). Mai et al. 2018 found that age, use of oral corticosteroids, and strenuous exercise were significantly associated with marked height loss (30). Fernihough & McGovern 2015 found that height loss among older English men and

For peer review only - http://bmjopen.bmj.com/site/about/guidelines.xhtml

women across a lifetime decreases by 2 cm to 4 cm on average (31). These numbers are similar to our findings, where we found an average decrease of 4.3 cm after 37 years.

Jain & Ma 2020 found that mid-aged and older Indonesian women exhibit considerable height loss despite being short to begin with, losing an average of 1.06 cm over 17 years (5). Ethnicity, working in the agricultural sector and availability of local health infrastructure were correlated with loss of height (5). Extreme height loss, greater than 3 cm, was associated with 8-10 percent lower lung function and grip strength and with an increased mortality (5). They also found that loss of height is higher among older age groups and taller individuals (5), which is in accordance with our results. A recent investigation demonstrated, using national register data linked to the same cohort as described here, that loss of height over 12 years predicted mortality from all-cause and cardiovascular disease (4). This is in line with the study of Jain & Ma (5). Such data is not widely available in prospective epidemiological studies.

As described, height loss is associated with poor health (1,4,5,8-10,30,31). Vertebral discs degenerate far earlier than other musculoskeletal tissues (1) and can lead to back pain and disability in the older adults (1) and in severe cases even vertebral fractures, which have been shown to have direct or indirect consequences such as decreased mobility, depression, hospitalisation, and mortality (22).

Panoramic dental radiography indices, cortical thickness, cortical erosion, and sparse trabeculation, have been suggested as screening tools for identifying risk groups with osteoporosis (16,17,24). Cortical erosion, assessed at dental practices, has been shown to be useful in the identification of undetected low bone mineral density among postmenopausal women (17). However, Okabe et al. 2008 found no significant correlations between cortical erosion assessments and the occurrence of fractures in a group of 80-years old Japanese men

For peer review only - http://bmjopen.bmj.com/site/about/guidelines.xhtml

and women (15). The difference in the results may be attributed to ethnicity-related anthropometric differences, like height, and the high frequencies of fragility fractures in Swedish and Norwegian women (27). Another difference is that Okabe et al. studied five years of fracture incidence whereas Jonasson et al investigated the same over ten years (15).

Mandibular cortical width is associated with general bone mineral density (16,32),but not with future osteoporotic fractures (28). Sparse trabeculation was shown to be associated with a twofold higher risk for future fracture in a younger group and with a three- to fourfold higher risk for future fracture in an older group (29). As far as we know, relationships between height loss and oral indices have not been examined before.

All bone structures undergo an alteration in the bone remodeling mechanism in connection with menopause, leading to greater bone loss than bone formation (33). The imbalance results in trabecular thinning, increase in intra-trabecular spacing, loss of connectivity in the meshwork and intra-cortical erosion, thereby leading to diminished bone strength and increased risk of fracture-(34,35). Taller women have thinner cortical bone (in the appendicular skeleton) and therefore a greater risk for fractures (36). The vertebral bone mass is largely trabecular with only a thin shell of cortical bone. The thin cortex together with the axial compressive load on the vertebra, distributed on fewer and thinner trabeculae, result in an increasing risk for fractures (37,38).

Strengths and limitations

The strengths are the prospective design, the population-based sample, the long follow-up, and the potential applicability of our findings to many settings, since many individuals visit their dentist at least every two years and radiographs are taken. The unique aspect of this study is its design, consisting of long-term panel data on bone structure and height. The main limitations are the fact that height loss reflects various conditions that may make accurate

For peer review only - http://bmjopen.bmj.com/site/about/guidelines.xhtml

height measures difficult. Moreover, participation rates became lower in the later years, and the drop out for different reasons, mortality included, probably differs between age groups, which might impact the mean height losses and the associations to covariates. The very low number of women with severely eroded cortex in the first period can explain why there was no significant association with height loss. This indicates that we cannot reach any reliable conclusion about the strength of correlation between cortical erosion and height loss in the age interval 38-50.

Implications of the findings

In our study population, the sparse trabeculation and the severe cortical erosion of the mandible could be seen to mimic the status of the vertebral trabeculae and intracortical erosion of the thin cortical shell of the vertebrae, which would, in turn, explain the potential height reduction. These two bone-related risk factors are important for the development of height loss as well as osteoporosis. A vertebral fracture is often asymptomatic, but it should not be ignored since it is a highly significant risk factor for future fractures. Therefore, using mandible status as a proxy and possible screening tool for the general status of vertebral trabeculae and intracortical bones is interesting for future health promotion.

However, implementation of new methods requires ethical considerations. Prevention has a long tradition in dentistry. Informing patients about their dental hygiene is an integral part of dental practice, whereas screening for osteoporosis/ risk of height loss is not. An ethical dilemma in health promotion is that risk information may have unintended negative consequences for people who are currently free of symptoms. This is demonstrated in a qualitative study of Hvas et al 2005 (39), whose interviews indicated that awareness of osteoporosis risk caused a feeling of worry in some postmenopausal women.

For peer review only - http://bmjopen.bmj.com/site/about/guidelines.xhtml

Visual assessments of the mandibular trabecular and cortical bone as conducted in this study are not routine in dental practice today, but simplified screening procedures using artificial intelligence may change this in the future (40). Many postmenopausal women are interested in their health, and if they ask their dentist about the radiographs taken, prudence is needed when the bone is not normal due to sparse trabeculation or severely eroded cortex.

CONCLUSION

Mandibular bone structure alterations such as severe cortical erosion and sparse trabeculation predicted height loss. They may therefore serve as proxy indicators when screening in the early phases of bone degenerative pathogenesis, signaling the ongoing bone remodeling and the need for further clinical attention to older women at risk for height loss.

Contributors: NNG and LL initiated the study. VS and GJ did the acquisition of data. NNG, LL, VS, GJ and ER contributed to the analysis and interpretation of data. VS and NNG did the statistical analyses. NNG wrote the first draft of the manuscript. NNG, LL, GJ, ER and VS critically revised the manuscript and approved the final version. LL obtained the funding.

Competing interest: None

Funding: This work was supported by grants from the Swedish Research Council for Health, Wing Life and Welfare (Forte, EpiLife 2016-1506), and the Swedish ALF-agreement (ALFGBG 720201).

Data sharing statement: No additional data available.

Ethics Approval: The Prospective Population Study of Women in Gothenburg has been approved by the Regional Ethics Review Board of the University of Gothenburg (*DNR 65-80,*

For peer review only

179-92, T453-04). The examinations comply with the Declaration of Helsinki and informed consent has been obtained from the subjects.

For peer review only

For peer review only - http://bmjopen.bmj.com/site/about/guidelines.xhtml

References

1. Hillier TA, Lui LY, Kado DM, LeBlanc ES, Vesco KK, Bauer DC, et al. Height loss in older women: risk of hip fracture and mortality independent of vertebral fractures. J Bone Miner Res 2012;27:153-159. doi: 10.1002/jbmr.558
2. Gavriilidou NN, Pihlsgard M, Elmstahl S. Anthropometric reference data for elderly Swedes and its disease-related pattern. Eur J Clin Nutr 2015;69:1066-1075. doi: 10.1038/ejcn.2015.73.
3. Pluskiewicz W, Adamczyk P, Drozdzowska B. The significance of height loss in postmenopausal women. The results from GO Study. Int J Clin Pract 2021;75:e14009. doi: 10.1111/ijcp.14009.
4. Klingberg S, Mehlig K, Dangol R, Björkelund C, Heitmann BL, Lissner L. Loss of height predicts total and cardiovascular mortality: a cohort study of northern European women. BMJ Open 2021;11:e049122. doi: 10.1136/bmjopen-2021-049122.
5. Jain U, Ma M. Height shrinkage, health and mortality among older adults: Evidence from Indonesia. Econ Hum Biol 2020;37:100863. doi: 10.1016/j.ehb.2020.100863.
6. Perissinotto E, Pisent C, Sergi G, Grigoletto F, Group IW. Anthropometric measurements in the elderly: age and gender differences. Br J Nutr 2002;87:177-186. doi: 10.1079/bjn2001487.
7. Gavriilidou NN, Pihlsgard M, Elmstahl S. High degree of BMI misclassification of malnutrition among Swedish elderly population: Age-adjusted height estimation using knee height and demispan. Eur J Clin Nutr 2015;69:565-571. doi: 10.1038/ejcn.2014.183.
8. Urban JP, Roberts S. Degeneration of the intervertebral disc. Arthritis Res Ther 2003;5:120-130. doi: 10.1186/ar629.

For peer review only

For peer review only - http://bmjopen.bmj.com/site/about/guidelines.xhtml

9. Wannamethee SG, Shaper AG, Lennon L, Whincup PH. Height loss in older men: associations with total mortality and incidence of cardiovascular disease. Arch Intern Med 2006;166:2546-2552. doi: 10.1001/archinte.166.22.2546.

10. Huang W, Lei X, Ridder G, Strauss J, Zhao Y. Health, Height, Height Shrinkage, and SES at Older Ages: Evidence from China. Am Econ J Appl Econ 2013;5:86-121. doi: 10.1257/app.5.2.86

11. Kanis JA. Assessment of fracture risk and its application to screening for postmenopausal osteoporosis: synopsis of a WHO report. WHO Study Group. Osteoporos Int 1994;4:368-381. doi: 10.1007/BF01622200

12. Jonasson G, Bankvall G, Kiliaridis S. Estimation of skeletal bone mineral density by means of the trabecular pattern of the alveolar bone, its interdental thickness, and the bone mass of the mandible. Oral Surg Oral Med Oral Pathol Oral Radiol Endod 2001;92:346-352. doi: 10.1067/moe.2001.116494.

13. Lindh C, Horner K, Jonasson G, Olsson P, Rohlin M, Jacobs R, et al. The use of visual assessment of dental radiographs for identifying women at risk of having osteoporosis: the OSTEODENT project. Oral Surg Oral Med Oral Pathol Oral Radiol Endod 2008;106:285-293. doi: 10.1016/j.tripleo.2007.09.008.

14. Lindh C, Petersson A, Rohlin M. Assessment of the trabecular pattern before endosseous implant treatment: diagnostic outcome of periapical radiography in the mandible. Oral Surg Oral Med Oral Pathol Oral Radiol Endod 1996;82:335-243. doi: 10.1016/s1079-2104(96)80363-5

15. Okabe S, Morimoto Y, Ansai T, Yoshioka I, Tanaka T, Taguchi A, et al. Assessment of the relationship between the mandibular cortex on panoramic radiographs and the risk of bone fracture and vascular disease in 80-year-olds. Oral Surg Oral Med Oral Pathol Oral Radiol Endod 2008;106:433-424. doi: 10.1016/j.tripleo.2007.09.013.

For peer review only - http://bmjopen.bmj.com/site/about/guidelines.xhtml

16. Taguchi A. Panoramic radiographs for identifying individuals with undetected osteoporosis. Japanese Dental Science Review 2009;45:109-120. doi.org/10.1016/j.jdsr.2009.05.001

17. Taguchi A, Ohtsuka M, Nakamoto T, Naito K, Tsuda M, Kudo Y, et al. Identification of post-menopausal women at risk of osteoporosis by trained general dental practitioners using panoramic radiographs. Dentomaxillofac Radiol 2007;36:149-154. doi: 10.1259/dmfr/31116116.

18. Lissner L, Skoog I, Andersson K, Beckman N, Sundh V, Waern M, et al. Participation bias in longitudinal studies: experience from the Population Study of Women in Gothenburg, Sweden. Scand J Prim Health Care 2003;21:242-247. doi: 10.1080/02813430310003309-1693.

19. Bengtsson C, Ahlqwist M, Andersson K, Bjorkelund C, Lissner L, Soderstrom M. The Prospective Population Study of Women in Gothenburg, Sweden, 1968-69 to 1992-93. A 24-year follow-up study with special reference to participation, representativeness, and mortality. Scand J Prim Health Care 1997;15:214-219. doi: 10.3109/02813439709035031.

20. Klemetti E, Kolmakov S, Kroger H. Pantomography in assessment of the osteoporosis risk group. Scand J Dent Res 1994;102:68-72. doi: 10.1111/j.1600-0722.1994.tb01156.x.

21. Sagiv M, Vogelaere PP, Soudry M, Ehrsam R. Role of physical activity training in attenuation of height loss through aging. Gerontology 2000;46:266-270. doi: 10.1159/000022170.

22. Xu W, Perera S, Medich D, Fiorito G, Wagner J, Berger LK, et al. Height loss, vertebral fractures, and the misclassification of osteoporosis. Bone 2011;48:307-311. doi: 10.1016/j.bone.2010.09.027.

For peer review only - http://bmjopen.bmj.com/site/about/guidelines.xhtml

23. Thornton MJ, Sedlak CA, Doheny MO. Height change and bone mineral density: revisited. Orthop Nurs 2004;23:315-320. doi: 10.1097/00006416-200409000-00007.

24. Jonasson G, Sundh V, Ahlqwist M, Hakeberg M, Bjorkelund C, Lissner L. A prospective study of mandibular trabecular bone to predict fracture incidence in women: a low-cost screening tool in the dental clinic. Bone 2011;49:873-879. doi: 10.1016/j.bone.2011.06.036.

25. Grimby G, Börjesson M, Jonsdottir IH, Schnohr P, Thelle DS, Saltin B. The "Saltin-Grimby Physical Activity Level Scale" and its application to health research. Scand J Med Sci Sports 2015;25 Suppl 4:119-25. doi: 10.1111/sms.12611

26. Kaufman JS, Cooper RS. Seeking Causal Explanations in Social Epidemiology. Am J Epidemiol 1999;150:113-120. doi: 10.1093/oxfordjournals.aje.a009969.

27. SBU. Osteoporos – prevention, diagnostik och behandling. En systematisk litteraturöversikt. Stockholm.: Statens beredning för medicinsk utvärdering (SBU). 2003.

28. Jonasson GB, Sundh V, Hakeberg M, Ahlqwist M, Lissner L, Hange D. Evaluation of clinical and radiographic indices as predictors of osteoporotic fractures: a 10-year longitudinal study. Oral Surg Oral Med Oral Pathol Oral Radiol 2018;125:487-494. doi: 10.1016/j.oooo.2017.11.009.

29. Sundh V, Hange D, Ahlqwist M, Hakeberg M, Lissner L, Jonasson G. FRAX and mandibular sparse trabeculation as fracture predictors: a longitudinal study from 1980 to 2002. Eur J Oral Sci 2017;125:135-140. doi: 10.1111/eos.12341.

30. Mai X, Marshall B, Hovey KM, Sperrazza J, Wactawski-Wende J. Risk factors for 5-year prospective height loss among postmenopausal women. Menopause 2018;25:883-889. doi: 10.1097/GME.0000000000001108.

For peer review only - http://bmjopen.bmj.com/site/about/guidelines.xhtml

31. Fernihough A, McGovern ME. Physical Stature Decline and the Health Status of the Elderly Population in England. Econ Hum Biol 2015;16:30-44. doi:10.1016/j.ehb.2013.12.010.

32. Devlin H, Horner K. Mandibular radiomorphometric indices in the diagnosis of reduced skeletal bone mineral density. Osteoporos Int 2002;13:373-378. doi: 10.1007/s001980200042.

33. Lerner UH. Bone remodeling in post-menopausal osteoporosis. J Dent Res 2006;85:584-595. doi: 10.1177/154405910608500703.

34. Bala Y, Zebaze R, Seeman E. Role of cortical bone in bone fragility. Curr Opin Rheumatol 2015;27(4):406-13. doi: 10.1097/BOR.0000000000000183.

35. Bjornerem A, Wang X, Bui M, Ghasem-Zadeh A, Hopper JL, Zebaze R, et al. Menopause-Related Appendicular Bone Loss is Mainly Cortical and Results in Increased Cortical Porosity. J Bone Miner Res 2018;33:598-605. doi: 10.1002/jbmr.3333.

36. Bjornerem A, Bui QM, Ghasem-Zadeh A, Hopper JL, Zebaze R, Seeman E. Fracture risk and height: an association partly accounted for by cortical porosity of relatively thinner cortices. J Bone Miner Res 2013;28:2017-2026. doi: 10.1002/jbmr.1934.

37. Adams MA, Hutton WC. The Mechanical Function of the Lumbar Apophyseal Joints. Spine 1983;8. doi: 10.1097/00007632-198304000-00017.

38. Giambini H, Salman Roghani R, Thoreson AR, Melton LJ, 3rd, An K-N, Gay RE. Lumbar trabecular bone mineral density distribution in patients with and without vertebral fractures: a case-control study. Eur Spine J 2014;23:1346-1353. doi: 10.1007/s00586-014-3205-2.

For peer review only - http://bmjopen.bmj.com/site/about/guidelines.xhtml

39. Hvas L, Reventlow S, Jensen HL, Malterud K. Awareness of risk of osteoporosis may cause uncertainty and worry in menopausal women. Scand J Public Health 2005;33:203–207. doi: 10.1080/14034940510005716

40. Jonasson G, Skoglund I, Rythén M. The rise and fall of the alveolar process: Dependency of teeth and metabolic aspects. Arch Oral Biol 2018;96:195-200. https://doi.org/10.1016/j.archoralbio.2018.09.016

For peer review only

Figure legend

Figure 1: Flow chart.

For peer review only

For peer review only - http://bmjopen.bmj.com/site/about/guidelines.xhtml

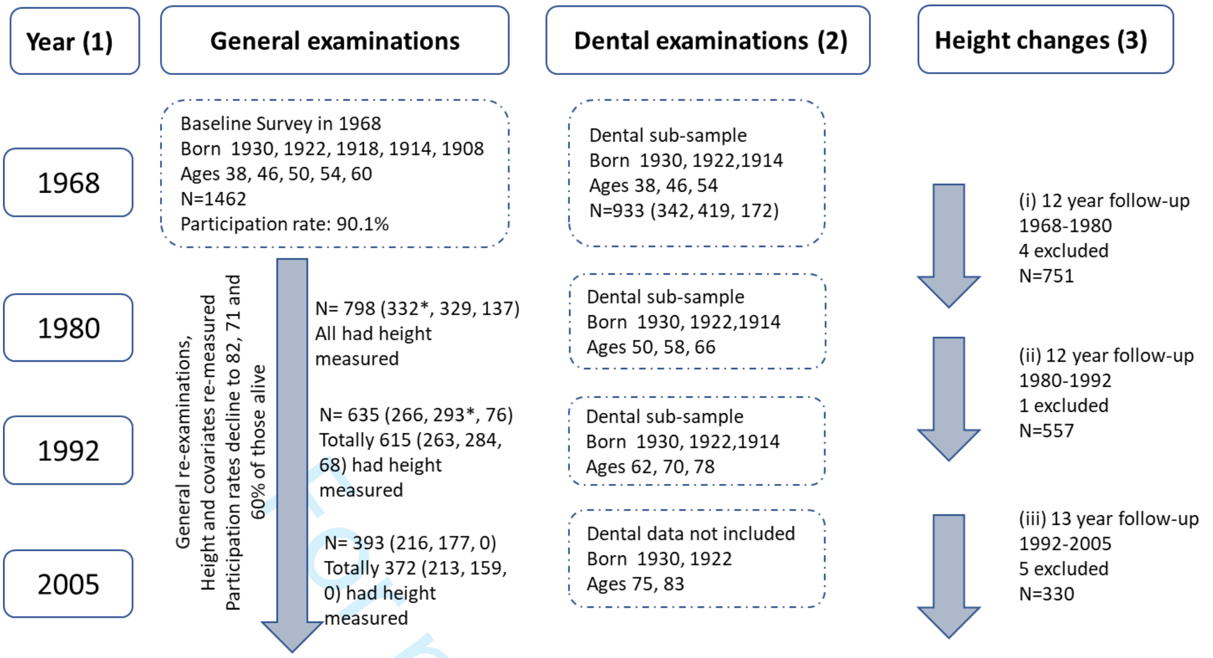

(1) Each examination period starting in September and ending in June of following year, i.e., 1968-69, 1980-81, 1992-93, 2005-06
(2) Dental examination conducted shortly after general examination in three of original age groups. Mandibular bone structure (cortical and trabecular bone) is the main independent variable
(3) Heights were measured at general examinations, and the change in height was estimated at 12- or 13-year intervals, after excluding any increases of over 2 cm

For peer review only

STROBE 2007 (v4) Statement—Checklist of items that should be included in reports of *cohort studies*

Section/Topic	Item #	Recommendation	Reported on page #
**Title and abstract**	1	(*a*) Indicate the study's design with a commonly used term in the title or the abstract	1 and 2
		(*b*) Provide in the abstract an informative and balanced summary of what was done and what was found	2
**Introduction**			
Background/rationale	2	Explain the scientific background and rationale for the investigation being reported	5
Objectives	3	State specific objectives, including any prespecified hypotheses	6
**Methods**			
Study design	4	Present key elements of study design early in the paper	2-5
Setting	5	Describe the setting, locations, and relevant dates, including periods of recruitment, exposure, follow-up, and data collection	2, 5
Participants	6	(*a*) Give the eligibility criteria, and the sources and methods of selection of participants. Describe methods of follow-up	5
		(*b*) For matched studies, give matching criteria and number of exposed and unexposed	-
Variables	7	Clearly define all outcomes, exposures, predictors, potential confounders, and effect modifiers. Give diagnostic criteria, if applicable	7, 8
Data sources/ measurement	8*	For each variable of interest, give sources of data and details of methods of assessment (measurement). Describe comparability of assessment methods if there is more than one group	*7, 8*
Bias	9	Describe any efforts to address potential sources of bias	11, 12
Study size	10	Explain how the study size was arrived at	7
Quantitative variables	11	Explain how quantitative variables were handled in the analyses. If applicable, describe which groupings were chosen and why	8
Statistical methods	12	(*a*) Describe all statistical methods, including those used to control for confounding	8
		(*b*) Describe any methods used to examine subgroups and interactions	8, 10
		(*c*) Explain how missing data were addressed	6
		(*d*) If applicable, explain how loss to follow-up was addressed	-
		(*e*) Describe any sensitivity analyses	-
**Results**			

For peer review only

Participants	13*	(a) Report numbers of individuals at each stage of study—eg numbers potentially eligible, examined for eligibility, confirmed eligible, included in the study, completing follow-up, and analysed	6, 7 +fig 1
		(b) Give reasons for non-participation at each stage	6, 7
		(c) Consider use of a flow diagram	Fig 1
Descriptive data	14*	(a) Give characteristics of study participants (eg demographic, clinical, social) and information on exposures and potential confounders	6, 7
		(b) Indicate number of participants with missing data for each variable of interest	Table 1
		(c) Summarise follow-up time (eg, average and total amount)	Fig 1 + tables
Outcome data	15*	Report numbers of outcome events or summary measures over time	5-8
Main results	16	(*a*) Give unadjusted estimates and, if applicable, confounder-adjusted estimates and their precision (eg, 95% confidence interval). Make clear which confounders were adjusted for and why they were included	Tables
		(*b*) Report category boundaries when continuous variables were categorized	Tables
		(*c*) If relevant, consider translating estimates of relative risk into absolute risk for a meaningful time period	-
Other analyses	17	Report other analyses done—eg analyses of subgroups and interactions, and sensitivity analyses	-
**Discussion**			
Key results	18	Summarise key results with reference to study objectives	10
**Limitations**			
Interpretation	20	Give a cautious overall interpretation of results considering objectives, limitations, multiplicity of analyses, results from similar studies, and other relevant evidence	14
Generalisability	21	Discuss the generalisability (external validity) of the study results	2, 11
**Other information**			
Funding	22	Give the source of funding and the role of the funders for the present study and, if applicable, for the original study on which the present article is based	15

*Give information separately for cases and controls in case-control studies and, if applicable, for exposed and unexposed groups in cohort and cross-sectional studies.

Note: An Explanation and Elaboration article discusses each checklist item and gives methodological background and published examples of transparent reporting. The STROBE checklist is best used in conjunction with this article (freely available on the Web sites of PLoS Medicine at http://www.plosmedicine.org/, Annals of Internal Medicine at http://www.annals.org/, and Epidemiology at http://www.epidem.com/). Information on the STROBE Initiative is available at www.strobe-statement.org.

For peer review only
